# A dataset of EEG and EOG from an auditory EOG-based communication system for patients in locked-in state

**DOI:** 10.1038/s41597-020-00789-4

**Published:** 2021-01-11

**Authors:** Andres Jaramillo-Gonzalez, Shizhe Wu, Alessandro Tonin, Aygul Rana, Majid Khalili Ardali, Niels Birbaumer, Ujwal Chaudhary

**Affiliations:** 1 Institute of Medical Psychology and Behavioral Neurobiology, University of Tübingen, Tübingen, Germany; 2 Wyss Center for Bio and Neuroengineering, Geneva, Switzerland; 3 Ospedale San Camillo, IRCCS, Venice, Italy

**Keywords:** Amyotrophic lateral sclerosis, Electroencephalography - EEG

## Abstract

The dataset presented here contains recordings of electroencephalogram (EEG) and electrooculogram (EOG) from four advanced locked-in state (LIS) patients suffering from ALS (amyotrophic lateral sclerosis). These patients could no longer use commercial eye-trackers, but they could still move their eyes and used the remnant oculomotor activity to select letters to form words and sentences using a novel auditory communication system. Data were recorded from four patients during a variable range of visits (from 2 to 10), each visit comprised of 3.22 ± 1.21 days and consisted of 5.57 ± 2.61 sessions recorded per day. The patients performed a succession of different sessions, namely, Training, Feedback, Copy spelling, and Free spelling. The dataset provides an insight into the progression of ALS and presents a valuable opportunity to design and improve assistive and alternative communication technologies and brain-computer interfaces. It might also help redefine the course of progression in ALS, thereby improving clinical judgement and treatment.

## Abstract (summary)

**Table:** 

Measurement(s)	eye movement measurement • brain measurement • muscle electrophysiology trait
Technology Type(s)	electrooculography • electroencephalography (EEG) • electromyography
Factor Type(s)	auditory stimuli • amyotrophic lateral sclerosis patient in locked-in state
Sample Characteristic - Organism	Homo sapiens

Machine-accessible metadata file describing the reported data: 10.6084/m9.figshare.13148762

## Background & Summary

Amyotrophic lateral sclerosis (ALS) is a neurodegenerative disorder that, in its final stages, paralyzes affected individuals impairing their ability to communicate^1–4^. Those patients with intact consciousness, voluntary eye movement control, who can blink their eyes or twitch their muscles are said to be in a locked-in state (LIS)^5,6^. Patients in LIS rely on eye-tracking based assistive and augmentative communication (AAC) technologies to communicate^7,8^. In the case of patients who survive attached to life-support systems, the progression of the disease ultimately destroys oculomotor control, leading to the loss of gaze-fixation and impeding the use of eye-tracking based communication technologies^9–11^. Nevertheless, even in the late stages of this condition, some remaining controllable muscles of the eyes continue to function for an unspecified length of time, which can be used to provide a means of communication to these patients^11,12^.

An auditory electrooculogram (EOG) based communication system^12^ was developed to provide a means of communication to ALS patients without gaze-fixation and who were unable to use the commercial AAC eye-tracking devices, but who had remnant oculomotor control to form words, phrases, and sentences using the system described in Tonin & Jaramillo-Gonzalez *et al*.^12^. Four ALS patients with progressively decreasing EOG signal amplitude in the range of ±200 μV to ±40 μV were able to select letters to construct words to form sentences and hence communicate freely using an auditory speller system. The auditory speller system is based on a binary system in which a patient is asked to respond to auditory questions by moving the eyes to say “yes” and not moving the eyes to say “no”. The system must use the auditory modality because, in these patients, vision is often impaired due to drying and necrosis of the cornea and the partly or fully paralyzed eye-muscles. The study design and paradigm are described in detail in the Methods section.

This data descriptor outlines the EEG and EOG recordings from four different patients recorded during their use of the auditory communication system, having first trained progressively, and then ultimately controlling the system to communicate. Electromyography (EMG) recordings are available for some sessions, according to the clinical conditions.

There have been other studies with similar goals, but only one has an available online dataset^13^, with different features. To our knowledge, in the available open-access specialized repositories^14–16^, there are no datasets with similar properties to the one described here. It must be emphasized that the data described here are both the EOG and EEG signals recorded with a dedicated set of electrodes for each type of signal simultaneously. These EEG and EOG data are recorded from patients with ALS in the most advanced stage, whose disease progression is not well defined and is, to a certain extent, unknown. The data highlight a phase in ALS where communication becomes difficult and gradually impossible with existing commercial AAC. It includes recordings of over the course of a year during which one of the users became unable to use the system because of disease progression. As a consequence, we believe that the study of this dataset might help towards improving the clinical definition of ALS in its very advanced state, the testing of hypotheses on the brain’s electrophysiological changes during this progression and evaluating the impact of advanced ALS on the cognitive state of the patients. Nevertheless, even though the data is quite specific, further investigation of the data can support novel clinical and therapeutic practices. It could help develop augmentative and alternative assistive communication technologies and brain-computer interfaces that can be generalized to other types of disorders and patients with pervasive communication deficits and motor impairments due to CNS damage, such as stroke or high spinal cord injury. Lastly, although the system can be considered successful in enabling communication, other analytical methods can still improve the system’s speed and efficiency, for example, offline testing of other feature extraction methods or testing and comparing the performance with different machine-learning methods to classify the patients’ response.

## Methods

The Internal Review Board of the Medical Faculty of the University of Tubingen approved the experiment reported in this study. The study was performed according to guidelines established by the Medical Faculty of the University of Tubingen. The patient, or the patient’s legal representative, gave informed consent with permission to publish the data. The clinical trial registration number is: ClinicalTrials.gov - Identifier: NCT02980380. The methods described here are complementary to an in-depth description of the results derived from this dataset that have been presented in related work^12^.

### Participating patients

Four ALS patients with amyotrophic lateral sclerosis with a functional rating scale revised (ALSFRS-R)^17^ score of 0 in the locked-in state (LIS) were visited on subsequent months starting from Feb 2018 to May 2019. Team members travelled to the patient’s home to perform the communication sessions, depending on the health status and convenience of the patient. The medical history of patients is described in our related work^12^. Every visit (V), lasted for a few days (D), during which the patient performed different session (S), as detailed in the Online-only Tables 1–4, with the precise dates of all the visits and details of the sessions.

### Auditory communication system

#### Prerequisites for performing the study

In agreement with the patients’ caretakers and considering the patients’ health and wellness and optimization of resources, it was established that the visits should be performed every two months approximately, with each visit no longer than four days. However, on some occasions the condition of the patients led to shorter visits, from three days to a single day. (see Online-only Tables 1–4). For each visit, guided by the same criteria of health and wellness of the patient, two team members transported all equipment and set up all systems in the patient’s home or accommodation.

Before the beginning of the study, at least 100 questions with known “yes” or “no” answers were formulated and recorded by a family member or caretaker in their own voice, in close proximity to the patient. Each question with a “yes” answer is paired with a similar question with “no” answer (e.g., “Paris is the capital of France” and “Berlin is the capital of France”). Each question is saved as an audio file with an explicit identifier, a question with a “yes” answer is saved with a 001_NUMBER identifier, and a question with “no” answer is saved with a 002_NUMBER identifier. The value of the label NUMBER is the same for a semantically paired sentence. The same procedure was repeated with biographical-related questions with at least 100 for every patient. Sentences are then stored on a laptop and accessed and played by the communication system during the sessions.

#### Study and paradigm

**Fig. 1:**
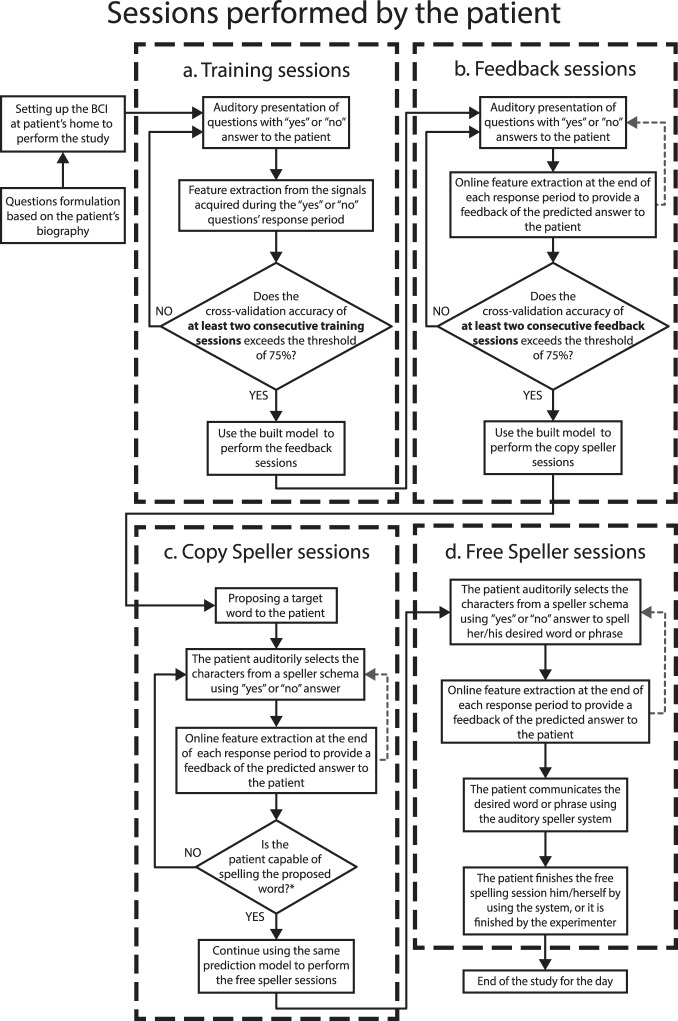
The procedure performed during a single day. The figure depicts the sequence of the types of sessions performed by patients and the criteria to progress from one type of session to the next. The patients first performed the Training sessions during which the patient learned to move his/her eyes to generate the signal to control the auditory communication system. At the end of the Training session, a classification model was built, and when the accuracy of the built model was greater than 75% the patients performed the feedback session. During the feedback sessions the patients were provided the feedback of their response, i.e., whether their answer was classified as “yes” or “no”. When the feedback accuracy exceeded 75% the patients first performed a copy speller session and then a free speller during which they could spell whatever they desired.

**Fig. 2:**
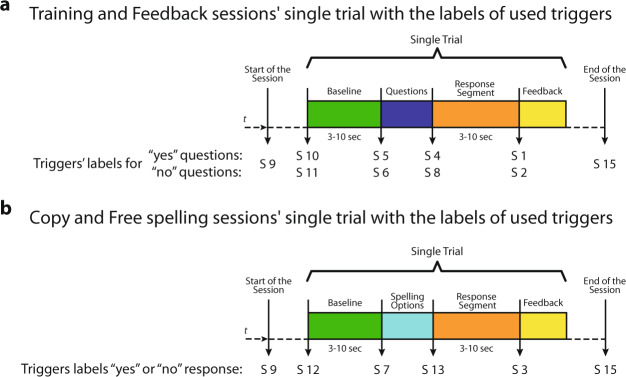
Different types of trials in the study. (**a**) Paradigm describing the sequence of events and sequence of the triggers’ labels used in a single trial for the Training and Feedback sessions. In these types of sessions, 20 questions with “yes” and “no” answers, known by the patient, are presented in a pseudo-random order. (**b**) Paradigm describing the sequence of events and sequence of the triggers’ labels used during a single trial for the Copy and Free spelling sessions. In these sessions, instead of questions, the patient is presented with options that allow him/her to navigate through his/her predetermined spelling scheme (e.g., sectors, letters). For both spelling sessions, the limit in the number of trials depends only on the patient’s attempts to spell the given target (i.e., Copy speller sessions) or her/his desired sentence (i.e., Open speller sessions). For any type of session recorded, the recording’s start and end are indicated by an “S 9” and an “S 15” trigger.

The study consisted of patients performing four different types of sessions, namely, Training, Feedback, Copy speller, and Free speller session, to train and enable the patient to employ an oculomotor strategy to control the spelling system successfully. During the visit, the patient performed different sessions, as depicted in Fig. 1. The patients developed a strategy to respond during successive trials to an auditory question (the questions previously recorded) by moving the eyes to say “yes” and by not moving the eyes to say “no”. To control the activities during the trials, specific paradigms were designed for the different sessions, as depicted in Fig. 2.

The different sessions performed by the patients are described below.

- Training session
  The study on a single day always started with Training sessions during which the patients were instructed to listen to a sequence of 20 personal questions consisting of 10 sentences with a “yes” answer and 10 with “no” answer, presented in pseudo-random order. After the system presents an auditory question, patients are asked to move their eyes to respond “yes” and not to move the eyes to respond “no” during a response time window. The duration of the response segment depended on the patient’s performance, i.e., if the patient could move his/her eye with ease, the duration was kept shorter and vice versa. Therefore, this window has a range from 3 to 10 seconds. For each Training session, the set of triggers indicating the sequence of events were recorded on the raw file, using the labels shown in Fig. 2a. Alongside, the system creates a questions sequence text file (Block_X_senlist.txt) that includes the list of identifiers of the presented audio/question files during the session (e.g., 001_13012a.wav), including also the label of the corresponding type of answer (“0” for sentences with “no” as an answer, and “1” for sentences with “yes” as an answer). The.txt lists are included inside the raw data folder structure, as described in the section Data Records. After at least two consecutive Training sessions with a classification accuracy result greater than 75%, the patient progresses to the Feedback sessions (see Fig. 1).
- Feedback session
  As in the Training session, the patients were presented with a sequence of a familiar question, but, at the end of the response segment, they were provided with auditory feedback as to whether their answer was recognized as “yes” or “no” by the system. For each Feedback session, the triggers indicating the events’ sequence were recorded on the raw file, using the labels shown in Fig. 2a. The system creates a sentence list (Block_X_senlist.txt) in the same way it was created for the Training sessions. In the case of Feedback sessions, in addition to the sequence of the questions text file, the system creates a result file (Date_result_f1_X.txt) listing the predicted results, i.e., “1” if the answer was recognized as “no”, “0” if the answer was recognized as “yes”, and “2” if the answer was unable to be classified by the system. The system gives the patient auditory feedback with the sentence: “Your answer was classified as yes/no”. Both.txt lists are also included inside the raw data folder structure, as described in the section Data Records. After at least two consecutive Feedback sessions with a classification accuracy result greater than 75%, the patient progresses to the Copy spelling sessions (see Fig. 1).
  The sequence of events and triggers (with their labels) for a single trial of the Training and Feedback sessions is depicted in Fig. 2a. Each of these trials consists of the segment of baseline (i.e., no sound presented), stimulus, during which the question is presented auditorily to the patients, followed by the segment of response time, in which the patient moves or does not move the eye according to his/her answer, and lastly the segment of feedback. For a Training session trail, the feedback is “thank you” to mark the end of the response while for a Feedback session trail, the feedback is “yes” or “no” depending on the answer classified by the system.
- Copy spelling session
  During the Copy spelling sessions, the patients were asked to spell a specific word described in our previous work^12^. For each Copy spelling session, the set of triggers indicating the sequence of events was recorded on the raw file, as shown in Fig. 2b. For the Spelling sessions, there are no questions sequence text files, but there are results files (Date_result_f1_X.txt) with the label of the predicted answer, listing the predicted results as “1” if the answer was recognized as “no”, “0” if the answer was recognized as “yes”, and “2” if the system was unable to classify the answer. The.txt lists are also included in the raw data folder structure described in the section Data Records.
- Free spelling session

After completing the Copy spelling session, the patients were asked to spell whatever he/she desired. For each Free spelling session, the set of triggers indicating the sequence of events were recorded on the raw file, as shown in Fig. 2b. As in the Copy spelling case, each Free spelling session created a result file (Date_result_f1_X.txt) using the same label code. The.txt lists are also included in the raw data folder structure described in the section Data Records.

The trials for the Copy and Free spelling sessions do not consist of the pre-recorded personal questions, but instead, of “yes”/”no” questions asking the patient whether to select or not, a particular letter, group of letters, or command, from his/her particular speller scheme^12^. Copy and Free spelling sessions differ in terms of the instruction given to the patient. During the Copy spelling sessions, the patient was asked to spell a specific word, while during the Free spelling sessions, the patient was asked to spell whatever he/she desired. Consequently, instead of being a fixed number, the number of trials in these sessions depends on the number of attempts performed by the patient to spell the given target (for the Copy speller sessions) or his/her desired sentence (for the Free speller sessions).

The sequence of events and triggers (with their labels) for a single trial for the Copy and Free spelling sessions is depicted in Fig. 2b. The trials consist of the segment of baseline (i.e., no sound presented), stimulus, where instead of questions the patient is presented with auditory options that allow him/her to navigate through his/her predetermined spelling scheme^12^ (e.g., sectors, characters, letters), followed by the response time segment in which the patient move or not move the eye according to his/her answer. Lastly, the feedback segment, during which depending on the answer classified by the system, “yes” or “no” auditory feedback is given to the patient.

Regardless of the session type, the recording time’s start and end are labeled by “S 9” and “S 15” triggers. During each trial, the sequence of events is presented to the patient and simultaneously, in a synchronized manner, a system of digital triggers is created by a Matlab script interacting with the V-Amp amplifier, to indicate the onset of each event in the time series. Both Fig. 2a,b show the sequence of triggers (their labels) as used in each trial. Information on the onset and labels of each event is also provided (see section Data Records).

We have to add that during the setting up of the system or the sessions’ execution, patients’ care and wellness were a high priority; therefore, under any request or signal of unease, sessions or even the day’s study were stopped.

#### System for data acquisition

The communication system is composed of the different elements described below.

- Laptop: The present setup uses a laptop with 8 GB RAM, Windows 7 operating system, and 3.3 GHz processor.
- EEG amplifier and recorder: For each session, EEG and EOG channels were recorded according to the 10-20 EEG electrode positioning system, with a 16 channel EEG amplifier (V-Amp DC, Brain Products, Germany) with Ag/AgCl active electrodes.
- EOG channels: at least four electrodes were recorded (positions SO1 and IO1 for vertical eye movement, and LO1 and LO2 for horizontal eye movement).
- EEG channels: at least seven channels located in central and prefrontal areas were recorded (exact locations per day in the Online-only Tables 1–4).
- EMG channels: on a limited number of sessions electrodes located on the chin of the patient or any other face muscle with assumed remaining function.

**Fig. 3:**
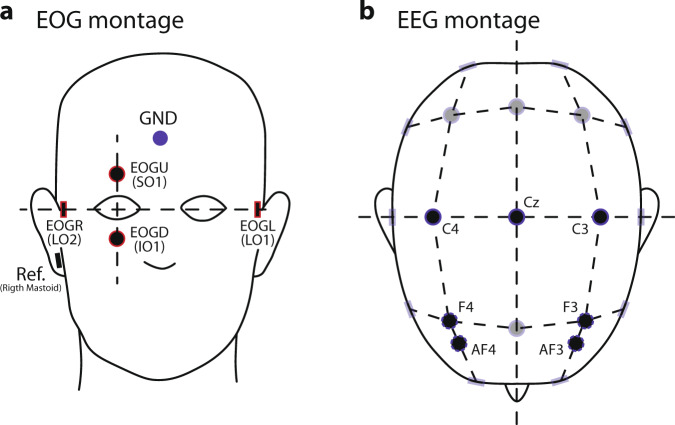
EOG and EEG setup. (**a**) Montage for the minimum number of EOG channels for each recorded session, using the locations LO1 (left cantus) and LO2 (right cantus) for horizontal eye movement, and SO1 (above superior orbit) and IO1 (below inferior orbit) for vertical eye movement. We used the labels EOGL, EOGR, EOGU, and EOGD, respectively, for the *online* study. (**b**) Montage for the minimum number of EEG electrodes for each recorded session, emphasizing the central motor (C4, Cz, C3) and prefrontal areas. In this latter case, the location of used electrodes might vary between F3 and F4, or Af4 and Af3. Nevertheless, the total number of electrodes might vary between days of the visits due to the patient’s wellness conditions. The exact number of electrodes and labels used can be verified in the Online-only Tables S1–S4.

All the channels were referenced to an electrode on the right mastoid and grounded to electrode FPz on the forehead. For the montage, electrode impedances were kept below 10 kΩ. The sampling frequency was 500 Hz. The standard montage for the minimum number of available EOG and EEG electrodes is specified in Fig. 3. The precise number and location of electrodes available for each session are detailed in the Online-only Tables 1–4, including recording EMG electrodes.

- Serial cable: This cable is used to connect the Laptop and the EEG amplifier to send the triggers with the custom Matlab code to mark the EEG-EOG recording with the different segments’ starting point.
- Loudspeakers: Loudspeakers connected to the laptop performs the function of delivering the audio stimuli to patients during the Training/Feedback/Copy spelling/Free spelling sessions, as described below.

## Data Records

### Raw data folders

The data stream was recorded directly from the EEG amplifier and stored with the proprietary BrainVision Recorder format^18,19^ during the sessions. According to the dongle key available during the visit, the data were stored in two possible formats, necessary to access and use BrainVision Recorder, 42% of data were recorded in *.ahdr and the rest 58% in *.vhdr format. For consistency here, we present the data in *.vdhr after converting the other 42% of *.ahdr format data also to *.vhdr format. Thus, as an output of this recording scheme, three output files per recording had the same name but different extension:

- Header file (*.vhdr), containing recording parameters and further meta-information, as the scaling factor necessary to convert the recorded raw amplitude to milivolts.
- Marker file (*.vmrk) describes the events and their onset during the data recording, in this case, the sequence of triggers.
- Raw EEG data file (*.eeg) is a binary file containing the EEG and EOG data and additional recorded signals.

Nevertheless, to assist with handling the unmodified raw data, we have used the BrainVision Analyzer^20^ software to export all the recordings to the more accessible.vhdr format, but without altering anyhow the content of the data itself.

**Fig. 4:**
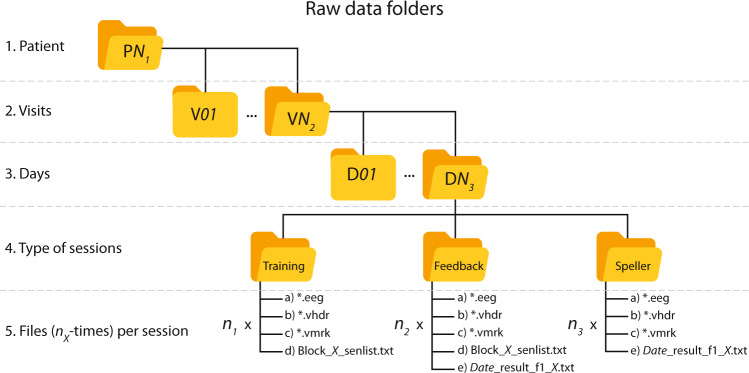
Raw data folder structure. Structure of nested folders containing the raw recordings of the study. According to the patient identifier, the upper level is the folder, which can be P*N*_*1*_ = 11,13,15 or 16. In the next level, V*N*_*2*_ indicates the total number of visits available for that patient, and inside it, D*N3* indicates the number of days that the visit lasted. Each day’s folder stores subfolders for the Training, Feedback, and spelling (that stores recordings from both the Copy and Free speller sessions). Each of these folders contains a set of files that are the outcome of a recorded session (detailed in the section Data Records), times the number that particular type of session (i.e., *n*_*1*_, *n*_*2*_, and *n*_*3*_) was respectively performed during the day.

For storing the raw data, a database was created using a nested structure of five levels (see Fig. 4), from the top:

1. Patient folder, where P*N*_*1*_ can be either P11, P13, P15, or P16.
2. Visits folder, where V*N*_*2*_ indicates the total number of visits available for each particular patient.
3. Day folder, where D*N*_*3*_ indicates the number of days that the particular visit lasted.
4. Type of sessions, where data has been separated according to the type of sessions. Training, Feedback, and Spelling sessions (consisting of both Copy and Free spelling sessions).

At the 5^th^ level, according to the type of session, there might be up to five types of files stored, times the number of that particular session recorded on the day, i.e., *n*_*1*_, *n*_*2*_, and *n*_*3*_ (see Fig. 4). Namely, the hosted files can be:

*.vhdr, the exported version of the *.ahdr file.
*.vmrk, the exported version of the *. amrk file.
*.eeg, that is a binary file with the recorded data.
- Block sentence list (Block_*X*_senlist.txt), where *X* is the counter of the number of ongoing sessions. This type of *.txt file was only created for Training and Feedback sessions.
- Result list (*Date*_result_f1_*X*.txt), with the *Date* in which the recording was made, and *X* is the counter of the ongoing sessions. This type of *.txt file was only created for Feedback and both Speller sessions.

### Matlab data fields

**Fig. 5:**
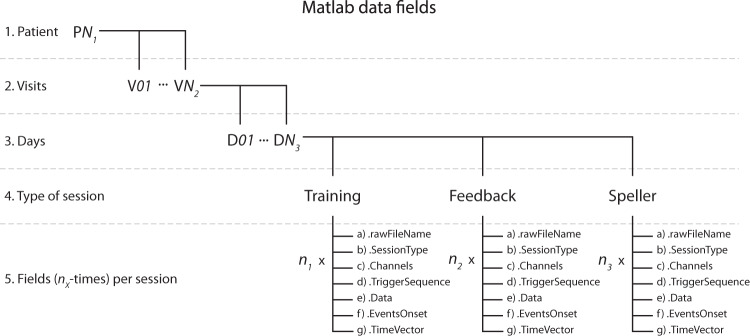
Matlab data fields structure. Nested structure elements containing the values and features of recordings from the study. According to the patient identifier, the upper level is the main structure, which can be P*N*_*1*_ = 11,13,15 or 16. In the next level, V*N*_*2*_ indicates the total number of visits available for that patient, and inside it, D*N3* indicates the number of days that the visit lasted. Inside each day, there are structures for the Training and Feedback sessions and the spelling sessions (containing recordings from both the Copy and Free speller sessions). Each of these contains a set of structures that result from exporting the *.vhdr raw files for each recorded session, times the number of that particular session type (i.e., *n*_*1*_, *n*_*2*_, and *n*_*3*_) was performed during the day. Read the Data Records section for details on the data exporting.

Additionally, another format has been chosen to present and share the data obtained from exporting the original raw files (details in the section Usage Notes). In this rectangular form, a Matlab variable is stored (*.mat), corresponding to the patient’s name, i.e., P11. In the variable, nested structures were created using a somehow similar architecture for the raw files, as detailed in Fig. 5. The levels, from upper to lower, are:

1. Patient structure, where P*N*_*1*_ can be either P11, P13, P15, or P16.
2. Visits structure, where V*N*_*2*_ indicates the total number of visits available for each particular patient.
3. Day structure, where D*N*_*3*_ indicates the number of days that the particular visit lasted.
4. Type of sessions structure, where data has been separated according to the type of sessions. Training, Feedback, and Spelling sessions (containing both copy and free spelling sessions).

**Fig. 6:**
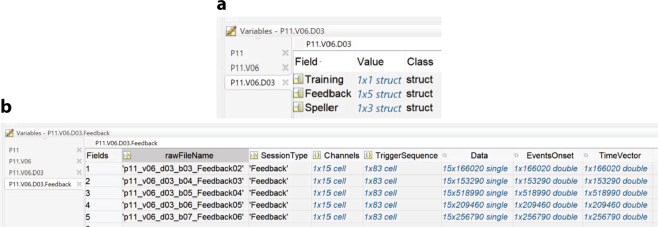
Example of a Matlab structure of the data using P11’s data. The figure illustrates the data structure using P11’s data from visit V06 and day D03. (**a**) Indicates the selection of patient variables and the data fields corresponding to a particular visit and day and inside it, the type and number of sessions performed on the given day. (**b**) Depicts the presence of different fields upon selecting a session type, in this case, the number of Feedback sessions performed by P11, upon selection of Field named Feedback, and their different elements, as shown in the figure. Read the Data Records section for the detailed description.

At the 5^th^ level, according to the type of session, there are seven fields stored, times the number of that particular session recorded on the day, i.e., *n*_*1*_, *n*_*2*_, and *n*_*3*_ (see Fig. 6). The hosted fields are:

- .rawFileName: character type variable with the name of the original raw file that was exported
- .SessionType: character type variable with the label of the type of session that the data belongs to
- .Channels: cell array with *1xK* dimensions, with *K* being the total number of EEG, EOG and EMG channels recorded, where each cell element is the label of a channel.
- .TriggerSequence: cell array with *1xM* dimensions, including the *Mth* events of all the trials recorded and the session as a sequence of triggers, with the labels indicated in Fig. 2., e,g., S 9, S 10, S 5, S 4, S 1, S 11, …, S 15
- .Data: a *KxR* dimensional matrix of numerical values, being *K* the number of channels recorded, and *R* the number of data points in the time domain of the recording, each element being the amplitude values of the recording. It is highly relevant to consider that the default amplitude of the recording needs to be multiplied for a scaling factor of 0.0488281 (±410 mV range in 24 bits) to convert to µV^21^. The scaling factor can be verified inside every *.vhdr file produced for every recording
- .EventsOnset; a *1xR* dimensional vector of numerical values, being *R* the number of data points in the time domain of the recording, and to each time point we have assigned the numerical value of the trigger labels (see Fig. 2) occurring at that time point, e.g., 9, 10, 5, 4, 1, 11, …, 15, and a value of zero otherwise. This vector aims to help quickly locate each event’s onset and nature in the time domain
- .TimeVector: a *1xR* dimensional vector of numerical values, being *R* the number of data points in the time domain of the recording, where an element of *R* indicates the time value in seconds of the recording.

As an example of the previous variable description, Fig. 6 illustrates the data structure using P11’s data from visit V01 and day D03.

All the datasets described in this section can be freely downloaded from the open access repository^22^.

## Technical Validation

The raw data referred to in this descriptor was recorded using a Brain Products V-Amp amplifier, without any type of hardware or software filter besides the physical instrumental restriction of the amplifier (wideband filter in the range of 0 Hz (DC) – 320 Hz or 4 kHz for the high-speed mode)^21^.

The raw data recorded with BrainVision Recorder software (v2.1.0) in *.ahdr, *.amrk, *.eeg formats were exported using BrainVision Analyzer software^20^ (v2.2.0) to obtain the formats^19^ *.vhdr, *.vmrk and *.eeg.

The data given as a Matlab format variable (*.mat) has been exported from the raw files taking advantage of the EEGLAB^23^ toolbox (https://sccn.ucsd.edu/eeglab/index.php, v2019.0) and the “bva-io” plugin (https://sccn.ucsd.edu/eeglab/plugin_uploader/plugin_list_all.php, v1.5.13), to save in the described variable structure desired features of the original raw file, as detailed in the Data Records section. No special parameter was used for exporting these data, and therefore we consider both raw and exported to the same values. Nevertheless, the amplitude of the recorded data (either.eeg or exported files using the “bva-io” plugin) is defined by the ADC bit resolution of the device, that is a ± 410 mV range in 24 bits, and therefore, the amplitude value needs to be multiplied by the scaling factor of 0.0488281^21^ to be converted to µV (microvolts) units. The resolution of each recording can be found per channel inside each *.vhdr given file.

The Matlab script used to export the raw files to Matlab variables (see Code Availability section) includes a deactivated code line that can be used to convert to µV the amplitude.

EOG electrodes were located and placed according to the standard 10-20 system with EEG neoprene caps (Neuroelectrics, Barcelona, Spain), inserted in the cap using plastic holders. Once the whole set of electrodes was in place, they were filled with SuperVisc electrolyte gel (Easycap, Germany, GmbH). Impedance was measured on the whole set using an ImpBox (Brain Products, Germany, GmbH), to achieve a target impedance of 10 KΩ. Researchers in charge of the study ensured that the recorded activity had the proper impedance and a clean signal for all the channels. Recordings are not affected by muscular or blinking artifacts, besides eye movements related to the patients’ intentions.

## Usage Notes

### Performance of the communication system

The communication system can present a question every nine seconds with an information transfer rate of 6.7 bits/min. The system’s optimal speed can be improved depending on the speller scheme’s design for each individual patient and the corpus of sentences stored for word prediction. Descriptive statistics on each patient’s performance can be found in the related publication^12^.

A minimal criterion for communicating using the system is the presence of eye-movements recordable with sate of the art EOG recording devices in the microvolt range. For one of the patients, the progression of the disease over the course of a year eventually prevented him from controlling his oculomotor activity. He was however capable of producing undifferentiated EOG activity with low amplitude in the range of ±30 μV which reached that minimal criterion. The other patients never arrived at such a total loss of control when the data described here was recorded. Therefore, the duration of the transition period to CLIS, and whether voluntary communication with non-invasive physiological recording technologies, as described here, will be possible in CLIS, is still a matter of future research.

### Date and time of the recordings

The original timestamp of the beginning of a recorded session can be found both inside the *.vhdr and the *.vmrk files, as the occurrence of the first marker in the recording. It can also be found as the timestamp of the sentence lists (Block_*X*_senlist.txt), or indicating the end of a session in the results text files (*Date*_result_f1_*X*.txt).

### Name of the raw files

During the study, files recorded with BrainVision Recorder software (v2.1.0) (i.e., *.vhdr, *.vmrk and *.eeg) were labeled by the experimenters, and therefore, human error or discrepancies might have been committed during the labeling process. To clarify any possible confusion, the Online-only Tables 1–4 include a set of columns that show the correspondence between the name of the raw file, the session’s sequence, and any *.txt files attached to it.

The text lists (Block_*X*_senlist.txt and *Date*_result_f1_*X*.txt) were created automatically by a Matlab script running during each session.

From the given recordings, either raw or Matlab fields, it can be noticed from the labels that some files or sessions are lacking. This is because during the visits, sessions belonging to another paradigm for a different and unrelated study were also recorded, and they have been deliberately removed from the actual data descriptor to focus on the auditory communication recordings. Removed files are indicated in the Online-only Tables 1–4. Additionally, a number of files were lost or corrupted; the precise number and sessions are indicated in Table 1 and Online-only Tables 1–4.

**Table 1:** The number of sessions in the dataset.

Patient	Visits	Days	Sessions		
			Training	Feedback	Speller
P11	9	27	68 (including 2 lost files)	56	26
P13	4	14	28	22	21
P15	2	7	7	16 (including 1 lost files)	18
P16	2	9	27	22	8

Detail of the number of visits and total days of the study, and the total number of different types of sessions recorded for each patient. Indicated in parenthesis are the numbers of lost recordings. Copy speller and free speller sessions are considered in the same column. A more detailed description of the days, dates, and sessions can be verified in the Online-only Tables 1–4.

### EEG locations and inconsistency

Working with patients who have critical health conditions means being completely dependent on their current (minute by minute) state. These limitations were considered in the design of the study. The number of EEG electrodes was limited by restrictions of accessibility of some scalp regions. Since the patients lie on their backs most of the time, it is impossible to access occipital areas.

Nevertheless, the most relevant restriction is the time constraint, that is, to place a minimal number of EEG electrodes in appropriate locations, in the minimum possible time, so to maximize the time available to work with the patient before tiredness or another need (for example, sucking of saliva) prevents them from participating in the study. Consequently, the montages of electrodes might be affected by inconsistency in EEG electrode locations, even for the same patient, and different visits, since it is always dependent on changing circumstances of health and time.

Therefore, the criterion we follow aims to reach with the minimal number of electrodes the greatest coverage of the prefrontal and mesial surfaces of the brain (besides the EOG electrodes), under the assumption that the cognitive activity implicated in the processing of these questions might elicit changes in the electrical activity of the aforementioned cortical regions.

Regardless of that, we managed to keep a constant number of seven EEG electrodes and four EOG electrodes for most of the patients, for most of the visits, as can be verified in the Online-only Tables 1–4.

### Audio files

The audio files (recorded questions) used in this research contains personal information of the patients and their relatives and consequently, to make these audio files fully open and public will compromise their identities. These data^24^ have been uploaded with restricted access, therefore any researcher or laboratory interested in accessing the data to perform the analysis will have to sign an identity protection agreement document provided as a “Data Use Agreement” Supplementary material with this manuscript.

## Supplementary information

Supplementary material

## Data Availability

The given Matlab data variables were obtained by exporting the raw files (i.e., *.vhdr, *.vmrk, and *.eeg) using the EEGLAB^23^ toolbox (v2019.1.0) and exporting the data using the “bva-io” plugin (v1.5.13). We wrote a short Matlab script (ExportingCode_vhdr2mat.m) to export and save the desired features of the recordings, as thoroughly detailed in the section Data Records. The code is included in the same repository as the rest of the data, and it is accompanied by a brief document (ExportingCode_vhdr2mat.docx) explaining details of the code.

## Online-only Tables

**Online-only Table 1:** Session to Raw File-Sentence List-Result List Correspondence for P11.

Patient	Visit	Day	Session	Date	Type of Session	Raw File Name (*.eeg)	Sentence List Name (*.txt)	Result List Name (*.txt)	Remark	Electrodes
P11	V01	D01	S01	24-Mar-18	Training	(Lost) p11d1b1	Block_1_senlist.txt	-	Lost Raw Files	AF4, F4, AF3, F3, Cz, C1, C2, EOGD, EOGL, EOGR
		D01	S02	24-Mar-18	Training	(Lost) p11d1b2	Block_2_senlist.txt	-	Lost Raw Files	AF4, F4, AF3, F3, Cz, C1, C2, EOGD, EOGL, EOGR
		D01	S03	24-Mar-18	Training	p11d1b3	Block_3_senlist.txt	-		AF4, F4, AF3, F3, Cz, C1, C2, EOGD, EOGL, EOGR
		D01	S04	24-Mar-18	Feedback	p11d1b4fb1	Block_4_senlist.txt	24-Mar-2018_result_f1_4.txt		AF4, F4, AF3, F3, Cz, C1, C2, EOGD, EOGL, EOGR
		D01	S05	24-Mar-18	Feedback	p11d1b5fb2	Block_5_senlist.txt	24-Mar-2018_result_f1_5.txt		AF4, F4, AF3, F3, Cz, C1, C2, EOGD, EOGL, EOGR
		D01	S06	24-Mar-18	Feedback	p11d1b6fb3Correct	(Lost-Corrupted) Block_6_senlist.txt	24-Mar-2018_result_f1_6.txt	Sentence List has an extra prediction (21 total)	AF4, F4, AF3, F3, Cz, C1, C2, EOGD, EOGL, EOGR
		D02	S01	25-Mar-18	Feedback	p11d2b1	(Lost) Block_1_senlist.txt	25-Mar-2018_result_f1_1.txt	Lost Sentence List	AF4, F4, AF3, F3, Cz, C1, C2, EOGD, EOGL, EOGR
		D02	S02	25-Mar-18	Feedback	p11d2b2fb2	Block_2_senlist.txt	25-Mar-2018_result_f1_2.txt		AF4, F4, AF3, F3, Cz, C1, C2, EOGD, EOGL, EOGR
		D02	S03	25-Mar-18	*					AF4, F4, AF3, F3, Cz, C1, C2, EOGD, EOGL, EOGR
		D03	S01	26-Mar-18	Feedback	p11d3b1fb1	Block_1_senlist.txt	26-Mar-2018_result_f1_1.txt	18 trials (w/o trigger 15), hardware error in initial 7 triggers	AF4, F4, AF3, F3, Cz, C1, C2, EOGD, EOGL, EOGR
		D03	S02	26-Mar-18	Feedback	p11d3b1fb2	Block_2_senlist.txt	26-Mar-2018_result_f1_2.txt		AF4, F4, AF3, F3, Cz, C1, C2, EOGD, EOGL, EOGR
		D03	S03	26-Mar-18	*					AF4, F4, AF3, F3, Cz, C1, C2, EOGD, EOGL, EOGR
		D03	S04	26-Mar-18	*					AF4, F4, AF3, F3, Cz, C1, C2, EOGD, EOGL, EOGR
	V02	D01	S01	2-May-18	Training	d1_0001	Block_1_senlist.txt	-		EOGU, EOGD, EOGR, EOGL, AF4, F4, AF3, F3, Cz, C1, C2
		D01	S02	2-May-18	Training	d1_0002	Block_2_senlist.txt	-		EOGU, EOGD, EOGR, EOGL, AF4, F4, AF3, F3, Cz, C1, C3
		D01	S03	2-May-18	Feedback	d1_0003	Block_3_senlist.txt	02-May-2018_result_f1_3.txt		EOGU, EOGD, EOGR, EOGL, AF4, F4, AF3, F3, Cz, C1, C4
		D01	S04	2-May-18	*					EOGU, EOGD, EOGR, EOGL, AF4, F4, AF3, F3, Cz, C1, C5
		D01	S05	2-May-18	*					EOGU, EOGD, EOGR, EOGL, AF4, F4, AF3, F3, Cz, C1, C6
		D02	S01	3-May-18	Feedback	d2_0001	Block_1_senlist.txt	(Corrupted) 03-May-2018_result_f1_1.txt	Result List is corrupted, has 26 predictions	EOGU, EOGD, EOGR, EOGL, AF4, F4, AF3, F3, Cz, C1, C7
		D02	S02	3-May-18	Feedback	d2_0002	Block_2_senlist.txt	03-May-2018_result_f1_2.txt		EOGU, EOGD, EOGR, EOGL, AF4, F4, AF3, F3, Cz, C1, C8
		D02	S03	3-May-18	Training	d2_0003	Block_3_senlist.txt	-		EOGU, EOGD, EOGR, EOGL, AF4, F4, AF3, F3, Cz, C1, C9
		D02	S04	3-May-18	Feedback	d2_0004	Block_4_senlist.txt	03-May-2018_result_f1_4.txt		EOGU, EOGD, EOGR, EOGL, AF4, F4, AF3, F3, Cz, C1, C10
		D03	S01	4-May-18	Training	d3_0001_ok	Block_1_senlist.txt	-		EOGU, EOGD, EOGR, EOGL, AF4, F4, AF3, F3, Cz, C1, C11
		D03	S02	4-May-18	Training	d3_0002	Block_2_senlist.txt	-		EOGU, EOGD, EOGR, EOGL, AF4, F4, AF3, F3, Cz, C1, C12
		D03	S03	4-May-18	Feedback	d3_0003	Block_3_senlist.txt	04-May-2018_result_f1_3.txt		EOGU, EOGD, EOGR, EOGL, AF4, F4, AF3, F3, Cz, C1, C13
		D03	S04	4-May-18	*					EOGU, EOGD, EOGR, EOGL, AF4, F4, AF3, F3, Cz, C1, C14
		D03	S05	4-May-18	*					EOGU, EOGD, EOGR, EOGL, AF4, F4, AF3, F3, Cz, C1, C15
		D04	S01	5-May-18	Training	d4_0001	Block_1_senlist.txt	-	Only 3 Trials	EOGU, EOGD, EOGR, EOGL, AF4, F4, AF3, F3, Cz, C1, C16
		D04	S02	5-May-18	Feedback	d4_0002	Block_2_senlist.txt	05-May-2018_result_f1_2.txt		EOGU, EOGD, EOGR, EOGL, AF4, F4, AF3, F3, Cz, C1, C17
		D04	S03	5-May-18	*					EOGU, EOGD, EOGR, EOGL, AF4, F4, AF3, F3, Cz, C1, C18
		D04	S04	5-May-18	*					EOGU, EOGD, EOGR, EOGL, AF4, F4, AF3, F3, Cz, C1, C19
	V04	D01	S01	21-Aug-18	Training (Motor Attempt)	p11_v04_d01_b01_trainingmotor	Block_1_senlist.txt	-		AF4, F2, F4, FC2, FC4, C2, Cz, Fz, AF3, F1, F3, FC1, FC3, C1, EOGR, EOGL
		D01	S02	21-Aug-18	Training (Motor Attempt)	p11_v04_d01_b02_trainingmotor	Block_2_senlist.txt	-		AF4, F2, F4, FC2, FC4, C2, Cz, Fz, AF3, F1, F3, FC1, FC3, C1, EOGR, EOGL
		D01	S03	21-Aug-18	Training (Motor Attempt)	p11_v04_d01_b03_trainingmotor	Block_3_senlist.txt	-		AF4, F2, F4, FC2, FC4, C2, Cz, Fz, AF3, F1, F3, FC1, FC3, C1, EOGR, EOGL
		D01	S04	21-Aug-18	Training (Motor Attempt)	p11_v04_d01_b04_trainingmotor	Block_4_senlist.txt	-		AF4, F2, F4, FC2, FC4, C2, Cz, Fz, AF3, F1, F3, FC1, FC3, C1, EOGR, EOGL
		D01	S05	21-Aug-18	Training (Motor Attempt)	p11_v04_d01_b05_trainingmotor	Block_5_senlist.txt	-		AF4, F2, F4, FC2, FC4, C2, Cz, Fz, AF3, F1, F3, FC1, FC3, C1, EOGR, EOGL
		D01	S06	21-Aug-18	Training (Motor Attempt)	p11_v04_d01_b06_trainingmotor	Block_6_senlist.txt	-		AF4, F2, F4, FC2, FC4, C2, Cz, Fz, AF3, F1, F3, FC1, FC3, C1, EOGR, EOGL
		D01	S07	21-Aug-18	Training (Motor Attempt)	p11_v04_d01_b07_trainingmotor	Block_7_senlist.txt	-		AF4, F2, F4, FC2, FC4, C2, Cz, Fz, AF3, F1, F3, FC1, FC3, C1, EOGR, EOGL
		D01	S08	21-Aug-18	Training (Motor Attempt)	p11_v04_d01_b08_trainingmotor	Block_8_senlist.txt	-		AF4, F2, F4, FC2, FC4, C2, Cz, Fz, AF3, F1, F3, FC1, FC3, C1, EOGR, EOGL
		D01	S09	21-Aug-18	Training (Motor Attempt)	p11_v04_d01_b09_trainingmotor	Block_9_senlist.txt	-		AF4, F2, F4, FC2, FC4, C2, Cz, Fz, AF3, F1, F3, FC1, FC3, C1, EOGR, EOGL
		D02	S01	22-Aug-18	Training	p11_v04_d02_b1_training	Block_1_senlist.txt	-		AF4, F2, F4, FC2, FC4, C2, Cz, Fz, AF3, F1, F3, FC1, FC3, C1, EOGR, EOGL
		D02	S02	22-Aug-18	Training	p11_v04_d02_b02_training	Block_2_senlist.txt	-		AF4, F2, F4, FC2, FC4, C2, Cz, Fz, AF3, F1, F3, FC1, FC3, C1, EOGR, EOGL
		D02	S03	22-Aug-18	Training	p11_v04_d02_b03_training	Block_3_senlist.txt	-		AF4, F2, F4, FC2, FC4, C2, Cz, Fz, AF3, F1, F3, FC1, FC3, C1, EOGR, EOGL
		D02	S04	22-Aug-18	Feedback	p11_v04_d02_b04_feedback	Block_4_senlist.txt	22-Aug-2018_result_f1_4.txt		AF4, F2, F4, FC2, FC4, C2, Cz, Fz, AF3, F1, F3, FC1, FC3, C1, EOGR, EOGL
		D02	S05	22-Aug-18	Training (Motor Attempt)	p11_v04_d02_b05_trainingmotor	Block_5_senlist.txt	-		AF4, F2, F4, FC2, FC4, C2, Cz, Fz, AF3, F1, F3, FC1, FC3, C1, EOGR, EOGL
		D02	S06	22-Aug-18	Training (Motor Attempt)	p11_v04_d02_b06_trainingmotor	Block_6_senlist.txt	-		AF4, F2, F4, FC2, FC4, C2, Cz, Fz, AF3, F1, F3, FC1, FC3, C1, EOGR, EOGL
		D02	S07	22-Aug-18	Training (Motor Attempt)	p11_v04_d02_b07_trainingmotor	Block_7_senlist.txt	-		AF4, F2, F4, FC2, FC4, C2, Cz, Fz, AF3, F1, F3, FC1, FC3, C1, EOGR, EOGL
		D02	S08	22-Aug-18	Training (Motor Attempt)	p11_v04_d02_b08_trainingmotor	Block_8_senlist.txt	-		AF4, F2, F4, FC2, FC4, C2, Cz, Fz, AF3, F1, F3, FC1, FC3, C1, EOGR, EOGL
		D02	S09	22-Aug-18	Training (Motor Attempt)	p11_v04_d02_b09_trainingmotor	Block_9_senlist.txt	-		AF4, F2, F4, FC2, FC4, C2, Cz, Fz, AF3, F1, F3, FC1, FC3, C1, EOGR, EOGL
		D02	S10	22-Aug-18	Training (Motor Attempt)	p11_v04_d02_b10_trainingmotor	Block_10_senlist.txt	-		AF4, F2, F4, FC2, FC4, C2, Cz, Fz, AF3, F1, F3, FC1, FC3, C1, EOGR, EOGL
		D03	S01	23-Aug-18	Training (Motor Attempt)	p11_v04_d03_b02_training	Block_1_senlist.txt	-		AF4, F2, F4, FC2, FC4, C2, Cz, Fz, AF3, F1, F3, FC1, FC3, C1, EOGR, EOGL
		D03	S02	23-Aug-18	Training (Motor Attempt)	p11_v04_d03_b03_training	Block_2_senlist.txt	-		AF4, F2, F4, FC2, FC4, C2, Cz, Fz, AF3, F1, F3, FC1, FC3, C1, EOGR, EOGL
		D03	S03	23-Aug-18	Training (Motor Attempt)	p11_v04_d03_b04_training	Block_3_senlist.txt	-		AF4, F2, F4, FC2, FC4, C2, Cz, Fz, AF3, F1, F3, FC1, FC3, C1, EOGR, EOGL
		D03	S04	23-Aug-18	Feedback (Motor Attempt)	p11_v04_d03_b05_feedback	Block_4_senlist.txt	23-Aug-2018_result_f1_4.txt		AF4, F2, F4, FC2, FC4, C2, Cz, Fz, AF3, F1, F3, FC1, FC3, C1, EOGR, EOGL
		D03	S05	23-Aug-18	Feedback (Motor Attempt)	p11_v04_d03_b06_feedback	Block_5_senlist.txt	23-Aug-2018_result_f1_5.txt		AF4, F2, F4, FC2, FC4, C2, Cz, Fz, AF3, F1, F3, FC1, FC3, C1, EOGR, EOGL
		D03	S06	23-Aug-18	Training (Motor Attempt)	p11_v04_d03_b07_training	Block_6_senlist.txt	-		AF4, F2, F4, FC2, FC4, C2, Cz, Fz, AF3, F1, F3, FC1, FC3, C1, EOGR, EOGL
		D03	S07	23-Aug-18	Training (Motor Attempt)	p11_v04_d03_b08_training	Block_7_senlist.txt	-		AF4, F2, F4, FC2, FC4, C2, Cz, Fz, AF3, F1, F3, FC1, FC3, C1, EOGR, EOGL
		D04	S01	24-Aug-18	Training	p11_v04_d04_b01_training	Block_1_senlist.txt	-		AF4, F2, F4, FC2, FC4, C2, Cz, Fz, AF3, F1, F3, FC1, FC3, C1, EOGR, EOGL
		D04	S02	24-Aug-18	Feedback	p11_v04_d04_b02_feedback	Block_2_senlist.txt	24-Aug-2018_result_f1_2.txt		AF4, F2, F4, FC2, FC4, C2, Cz, Fz, AF3, F1, F3, FC1, FC3, C1, EOGR, EOGL
		D04	S03	24-Aug-18	*					AF4, F2, F4, FC2, FC4, C2, Cz, Fz, AF3, F1, F3, FC1, FC3, C1, EOGR, EOGL
		D04	S04	24-Aug-18	*					AF4, F2, F4, FC2, FC4, C2, Cz, Fz, AF3, F1, F3, FC1, FC3, C1, EOGR, EOGL
		D04	S05	24-Aug-18	*					AF4, F2, F4, FC2, FC4, C2, Cz, Fz, AF3, F1, F3, FC1, FC3, C1, EOGR, EOGL
		D04	S06	24-Aug-18	*					AF4, F2, F4, FC2, FC4, C2, Cz, Fz, AF3, F1, F3, FC1, FC3, C1, EOGR, EOGL
		D04	S07	24-Aug-18	*					AF4, F2, F4, FC2, FC4, C2, Cz, Fz, AF3, F1, F3, FC1, FC3, C1, EOGR, EOGL
		D04	S08	24-Aug-18	*					AF4, F2, F4, FC2, FC4, C2, Cz, Fz, AF3, F1, F3, FC1, FC3, C1, EOGR, EOGL
		D04	S09	24-Aug-18	*					AF4, F2, F4, FC2, FC4, C2, Cz, Fz, AF3, F1, F3, FC1, FC3, C1, EOGR, EOGL
		D04	S10	24-Aug-18	*					AF4, F2, F4, FC2, FC4, C2, Cz, Fz, AF3, F1, F3, FC1, FC3, C1, EOGR, EOGL
		D04	S11	24-Aug-18	*					AF4, F2, F4, FC2, FC4, C2, Cz, Fz, AF3, F1, F3, FC1, FC3, C1, EOGR, EOGL
		D04	S12	24-Aug-18	*					AF4, F2, F4, FC2, FC4, C2, Cz, Fz, AF3, F1, F3, FC1, FC3, C1, EOGR, EOGL
		D04	S13	24-Aug-18	*					AF4, F2, F4, FC2, FC4, C2, Cz, Fz, AF3, F1, F3, FC1, FC3, C1, EOGR, EOGL
		D04	S14	24-Aug-18	*					AF4, F2, F4, FC2, FC4, C2, Cz, Fz, AF3, F1, F3, FC1, FC3, C1, EOGR, EOGL
		D04	S15	24-Aug-18	*					AF4, F2, F4, FC2, FC4, C2, Cz, Fz, AF3, F1, F3, FC1, FC3, C1, EOGR, EOGL
	V05	D01	S01	19-Sep-18	Training	p11_v05_d01_b02_training_01	Block_1_senlist.txt	-		AF4, F2, F4, FC2, FC4, Cz, Fz, AF3, F1, F3, FC1, FC3, EOGU, EOGD, EOGR, EOGL
		D01	S02	19-Sep-18	Training	p11_v05_d01_b03_training_02	Block_2_senlist.txt	-		AF4, F2, F4, FC2, FC4, Cz, Fz, AF3, F1, F3, FC1, FC3, EOGU, EOGD, EOGR, EOGL
		D01	S03	19-Sep-18	Training	p11_v05_d01_b04_training_03	Block_3_senlist.txt	-		AF4, F2, F4, FC2, FC4, Cz, Fz, AF3, F1, F3, FC1, FC3, EOGU, EOGD, EOGR, EOGL
		D01	S04	19-Sep-18	Feedback	p11_v05_d01_b05_feedback_04	Block_4_senlist.txt	19-Sep-2018_result_f1_4.txt		AF4, F2, F4, FC2, FC4, Cz, Fz, AF3, F1, F3, FC1, FC3, EOGU, EOGD, EOGR, EOGL
		D01	S05	19-Sep-18	Feedback	p11_v05_d01_b06_feedback_05	Block_5_senlist.txt	19-Sep-2018_result_f1_5.txt		AF4, F2, F4, FC2, FC4, Cz, Fz, AF3, F1, F3, FC1, FC3, EOGU, EOGD, EOGR, EOGL
		D02	S01	20-Sep-18	Training	p11_v05_d02_b02_training01	Block_1_senlist.txt	-		AF4, F2, F4, FC2, FC4, Cz, Fz, AF3, F1, F3, FC1, FC3, EOGU, EOGD, EOGR, EOGL
		D02	S02	20-Sep-18	Feedback	p11_v05_d02_b03_feedback02	Block_2_senlist.txt	20-Sep-2018_result_f1_2.txt		AF4, F2, F4, FC2, FC4, Cz, Fz, AF3, F1, F3, FC1, FC3, EOGU, EOGD, EOGR, EOGL
		D02	S03	20-Sep-18	*					AF4, F2, F4, FC2, FC4, Cz, Fz, AF3, F1, F3, FC1, FC3, EOGU, EOGD, EOGR, EOGL
		D02	S04	20-Sep-18	*					AF4, F2, F4, FC2, FC4, Cz, Fz, AF3, F1, F3, FC1, FC3, EOGU, EOGD, EOGR, EOGL
		D02	S05	20-Sep-18	*					AF4, F2, F4, FC2, FC4, Cz, Fz, AF3, F1, F3, FC1, FC3, EOGU, EOGD, EOGR, EOGL
		D02	S06	20-Sep-18	*					AF4, F2, F4, FC2, FC4, Cz, Fz, AF3, F1, F3, FC1, FC3, EOGU, EOGD, EOGR, EOGL
		D02	S07	20-Sep-18	*					AF4, F2, F4, FC2, FC4, Cz, Fz, AF3, F1, F3, FC1, FC3, EOGU, EOGD, EOGR, EOGL
		D03	S01	21-Sep-18	Training	p11_v05_d03_b02_training01	Block_1_senlist.txt	-		AF4, F2, F4, FC2, FC4, Cz, Fz, AF3, F1, F3, FC1, FC3, EOGU, EOGD, EOGR, EOGL
		D03	S02	21-Sep-18	Feedback	p11_v05_d03_b03_feedback02	Block_2_senlist.txt	21-Sep-2018_result_f1_2.txt		AF4, F2, F4, FC2, FC4, Cz, Fz, AF3, F1, F3, FC1, FC3, EOGU, EOGD, EOGR, EOGL
		D03	S03	21-Sep-18	*					AF4, F2, F4, FC2, FC4, Cz, Fz, AF3, F1, F3, FC1, FC3, EOGU, EOGD, EOGR, EOGL
		D03	S04	21-Sep-18	*					AF4, F2, F4, FC2, FC4, Cz, Fz, AF3, F1, F3, FC1, FC3, EOGU, EOGD, EOGR, EOGL
		D03	S05	21-Sep-18	*					AF4, F2, F4, FC2, FC4, Cz, Fz, AF3, F1, F3, FC1, FC3, EOGU, EOGD, EOGR, EOGL
		D03	S06	21-Sep-18	*					AF4, F2, F4, FC2, FC4, Cz, Fz, AF3, F1, F3, FC1, FC3, EOGU, EOGD, EOGR, EOGL
		D03	S07	21-Sep-18	*					AF4, F2, F4, FC2, FC4, Cz, Fz, AF3, F1, F3, FC1, FC3, EOGU, EOGD, EOGR, EOGL
		D03	S08	21-Sep-18	*					AF4, F2, F4, FC2, FC4, Cz, Fz, AF3, F1, F3, FC1, FC3, EOGU, EOGD, EOGR, EOGL
		D04	S01	22-Sep-18	Training	p11_v05_d04_b02_training01	Block_1_senlist.txt	-		AF4, F2, F4, FC2, FC4, Cz, Fz, AF3, F1, F3, FC1, FC3, EOGU, EOGD, EOGR, EOGL
		D04	S02	22-Sep-18	Feedback	p11_v05_d04_b03_feedback02	Block_2_senlist.txt	22-Sep-2018_result_f1_2.txt		AF4, F2, F4, FC2, FC4, Cz, Fz, AF3, F1, F3, FC1, FC3, EOGU, EOGD, EOGR, EOGL
		D04	S03	22-Sep-18	*					AF4, F2, F4, FC2, FC4, Cz, Fz, AF3, F1, F3, FC1, FC3, EOGU, EOGD, EOGR, EOGL
		D04	S04	22-Sep-18	*					AF4, F2, F4, FC2, FC4, Cz, Fz, AF3, F1, F3, FC1, FC3, EOGU, EOGD, EOGR, EOGL
		D04	S05	22-Sep-18	*					AF4, F2, F4, FC2, FC4, Cz, Fz, AF3, F1, F3, FC1, FC3, EOGU, EOGD, EOGR, EOGL
	V06	D01	S01	5-Nov-18	Training	p11_v06_d01_b02_Training01	Block_1_senlist.txt	-		F2, AF4, FC2, F4, C4, C2, Cz, C1, FC1, F3, F1, AF3, EOGU, EOGR, EOGL
		D01	S02	5-Nov-18	Training	p11_v06_d01_b03_Training02	Block_2_senlist.txt	-		F2, AF4, FC2, F4, C4, C2, Cz, C1, FC1, F3, F1, AF3, EOGU, EOGR, EOGL
		D01	S03	5-Nov-18	Feedback	p11_v06_d01_b04_Feedback03	Block_3_senlist.txt	05-Nov-2018_result_f1_3.txt		F2, AF4, FC2, F4, C4, C2, Cz, C1, FC1, F3, F1, AF3, EOGU, EOGR, EOGL
		D01	S04	5-Nov-18	Feedback	p11_v06_d01_b05_Feedback04	Block_4_senlist.txt	05-Nov-2018_result_f1_4.txt		F2, AF4, FC2, F4, C4, C2, Cz, C1, FC1, F3, F1, AF3, EOGU, EOGR, EOGL
		D01	S05	5-Nov-18	Feedback	p11_v06_d01_b06_Feedback05	Block_5_senlist.txt	05-Nov-2018_result_f1_5.txt		F2, AF4, FC2, F4, C4, C2, Cz, C1, FC1, F3, F1, AF3, EOGU, EOGR, EOGL
		D01	S06	5-Nov-18	*					F2, AF4, FC2, F4, C4, C2, Cz, C1, FC1, F3, F1, AF3, EOGU, EOGR, EOGL
		D01	S07	5-Nov-18	Speller	p11_v06_d01_b08_Speller07	-	05-Nov-2018_result_f1_7.txt	5 trials (w/o trigger 15)	F2, AF4, FC2, F4, C4, C2, Cz, C1, FC1, F3, F1, AF3, EOGU, EOGR, EOGL
		D02	S01	6-Nov-18	Training	p11_v06_d02_b02_Training01	Block_1_senlist.txt	-		F2, AF4, FC2, F4, C4, C2, Cz, C1, FC1, F3, F1, AF3, EOGU, EOGR, EOGL
		D02	S02	6-Nov-18	Training	p11_v06_d02_b03_Training02	Block_2_senlist.txt	-		F2, AF4, FC2, F4, C4, C2, Cz, C1, FC1, F3, F1, AF3, EOGU, EOGR, EOGL
		D02	S03	6-Nov-18	Feedback	p11_v06_d02_b04_Feedback03	Block_3_senlist.txt	06-Nov-2018_result_f1_3.txt		F2, AF4, FC2, F4, C4, C2, Cz, C1, FC1, F3, F1, AF3, EOGU, EOGR, EOGL
		D02	S04	6-Nov-18	Feedback	p11_v06_d02_b05_Feedback04	Block_4_senlist.txt	06-Nov-2018_result_f1_4.txt		F2, AF4, FC2, F4, C4, C2, Cz, C1, FC1, F3, F1, AF3, EOGU, EOGR, EOGL
		D02	S05	6-Nov-18	*					F2, AF4, FC2, F4, C4, C2, Cz, C1, FC1, F3, F1, AF3, EOGU, EOGR, EOGL
		D02	S06	6-Nov-18	*					F2, AF4, FC2, F4, C4, C2, Cz, C1, FC1, F3, F1, AF3, EOGU, EOGR, EOGL
		D02	S07	6-Nov-18	Speller	p11_v06_d02_b08_Speller07	-	06-Nov-2018_result_f1_7.txt	7 trials (w/o trigger 15)	F2, AF4, FC2, F4, C4, C2, Cz, C1, FC1, F3, F1, AF3, EOGU, EOGR, EOGL
		D02	S08	6-Nov-18	Speller	p11_v06_d02_b09_Speller08	-	06-Nov-2018_result_f1_8.txt	11 trials (w/o trigger 15)	F2, AF4, FC2, F4, C4, C2, Cz, C1, FC1, F3, F1, AF3, EOGU, EOGR, EOGL
		D02	S09	6-Nov-18	Speller	p11_v06_d02_b10_Speller09	-	06-Nov-2018_result_f1_9.txt	23 trials (w/o trigger 15)	F2, AF4, FC2, F4, C4, C2, Cz, C1, FC1, F3, F1, AF3, EOGU, EOGR, EOGL
		D02	S10	6-Nov-18	Speller	p11_v06_d02_b11_Speller10	-	06-Nov-2018_result_f1_10.txt	35 trials (w/o trigger 15)	F2, AF4, FC2, F4, C4, C2, Cz, C1, FC1, F3, F1, AF3, EOGU, EOGR, EOGL
		D03	S01	7-Nov-18	Training	p11_v06_d03_b02_Training01	Block_1_senlist.txt	-		F2, AF4, FC2, F4, C4, C2, Cz, C1, FC1, F3, F1, AF3, EOGU, EOGR, EOGL
		D03	S02	7-Nov-18	Feedback	p11_v06_d03_b03_Feedback02	Block_2_senlist.txt	07-Nov-2018_result_f1_2.txt		F2, AF4, FC2, F4, C4, C2, Cz, C1, FC1, F3, F1, AF3, EOGU, EOGR, EOGL
		D03	S03	7-Nov-18	Feedback	p11_v06_d03_b04_Feedback03	Block_3_senlist.txt	07-Nov-2018_result_f1_3.txt		F2, AF4, FC2, F4, C4, C2, Cz, C1, FC1, F3, F1, AF3, EOGU, EOGR, EOGL
		D03	S04	7-Nov-18	Feedback	p11_v06_d03_b05_Feedback04	Block_4_senlist.txt	07-Nov-2018_result_f1_4.txt		F2, AF4, FC2, F4, C4, C2, Cz, C1, FC1, F3, F1, AF3, EOGU, EOGR, EOGL
		D03	S05	7-Nov-18	Feedback	p11_v06_d03_b06_Feedback05	Block_5_senlist.txt	07-Nov-2018_result_f1_5.txt		F2, AF4, FC2, F4, C4, C2, Cz, C1, FC1, F3, F1, AF3, EOGU, EOGR, EOGL
		D03	S06	7-Nov-18	Feedback	p11_v06_d03_b07_Feedback06	Block_6_senlist.txt	07-Nov-2018_result_f1_6.txt		F2, AF4, FC2, F4, C4, C2, Cz, C1, FC1, F3, F1, AF3, EOGU, EOGR, EOGL
		D03	S07	7-Nov-18	*					F2, AF4, FC2, F4, C4, C2, Cz, C1, FC1, F3, F1, AF3, EOGU, EOGR, EOGL
		D03	S08	7-Nov-18	*					F2, AF4, FC2, F4, C4, C2, Cz, C1, FC1, F3, F1, AF3, EOGU, EOGR, EOGL
		D03	S09	7-Nov-18	Speller	p11_v06_d03_b10_Speller09	-	07-Nov-2018_result_f1_9.txt	Session finishing w/o trigger 15	F2, AF4, FC2, F4, C4, C2, Cz, C1, FC1, F3, F1, AF3, EOGU, EOGR, EOGL
		D03	S10	7-Nov-18	Speller	p11_v06_d03_b11_Speller10	-	07-Nov-2018_result_f1_10.txt		F2, AF4, FC2, F4, C4, C2, Cz, C1, FC1, F3, F1, AF3, EOGU, EOGR, EOGL
		D03	S11	7-Nov-18	Speller	p11_v06_d03_b12_Speller11	-	07-Nov-2018_result_f1_11.txt		F2, AF4, FC2, F4, C4, C2, Cz, C1, FC1, F3, F1, AF3, EOGU, EOGR, EOGL
	V07	D02	S01	12-Dec-18	Training	p11_v07_d02_b02_training01	Block_1_senlist.txt	-		F2, F4, FC2, FC4, C2, Cz, C1, FC1, FC3, F1, F3, EOGU, EOGD, EOGR, EOGL
		D02	S02	12-Dec-18	Training	p11_v07_d02_b03_training02	Block_2_senlist.txt	-		F2, F4, FC2, FC4, C2, Cz, C1, FC1, FC3, F1, F3, EOGU, EOGD, EOGR, EOGL
		D02	S03	12-Dec-18	Feedback	p11_v07_d02_b04_feedback03	Block_3_senlist.txt	12-Dec-2018_result_f1_3.txt		F2, F4, FC2, FC4, C2, Cz, C1, FC1, FC3, F1, F3, EOGU, EOGD, EOGR, EOGL
		D02	S04	12-Dec-18	Feedback	p11_v07_d02_b05_feedback04	Block_4_senlist.txt	12-Dec-2018_result_f1_4.txt		F2, F4, FC2, FC4, C2, Cz, C1, FC1, FC3, F1, F3, EOGU, EOGD, EOGR, EOGL
		D02	S05	12-Dec-18	*					F2, F4, FC2, FC4, C2, Cz, C1, FC1, FC3, F1, F3, EOGU, EOGD, EOGR, EOGL
		D02	S06	12-Dec-18	Speller	p11_v07_d02_b07_speller06	-	12-Dec-2018_result_f1_6.txt		F2, F4, FC2, FC4, C2, Cz, C1, FC1, FC3, F1, F3, EOGU, EOGD, EOGR, EOGL
		D02	S07	12-Dec-18	Speller	p11_v07_d02_b08_speller07	-	12-Dec-2018_result_f1_7.txt		F2, F4, FC2, FC4, C2, Cz, C1, FC1, FC3, F1, F3, EOGU, EOGD, EOGR, EOGL
		D02	S08	12-Dec-18	Speller	p11_v07_d02_b09_speller08	-	12-Dec-2018_result_f1_8.txt	33 trials (w/o trigger 15)	F2, F4, FC2, FC4, C2, Cz, C1, FC1, FC3, F1, F3, EOGU, EOGD, EOGR, EOGL
		D02	S09	12-Dec-18	Speller	p11_v07_d02_b10_speller09	-	12-Dec-2018_result_f1_9.txt	33 trials (w/o trigger 15)	F2, F4, FC2, FC4, C2, Cz, C1, FC1, FC3, F1, F3, EOGU, EOGD, EOGR, EOGL
		D02	S10	12-Dec-18	Speller	p11_v07_d02_b11_speller10	-	12-Dec-2018_result_f1_10.txt		F2, F4, FC2, FC4, C2, Cz, C1, FC1, FC3, F1, F3, EOGU, EOGD, EOGR, EOGL
		D02	S11	12-Dec-18	Speller	p11_v07_d02_b12_speller11	-	12-Dec-2018_result_f1_11.txt		F2, F4, FC2, FC4, C2, Cz, C1, FC1, FC3, F1, F3, EOGU, EOGD, EOGR, EOGL
		D03	S01	13-Dec-18	Training	p11_v07_d03_b02_training01	Block_1_senlist.txt	-		F2, F4, FC2, FC4, C2, Cz, C1, FC1, FC3, F1, F3, EOGU, EOGD, EOGR, EOGL
		D03	S02	13-Dec-18	Training	p11_v07_d03_b03_training02	Block_2_senlist.txt	-		F2, F4, FC2, FC4, C2, Cz, C1, FC1, FC3, F1, F3, EOGU, EOGD, EOGR, EOGL
		D03	S03	13-Dec-18	Feedback	p11_v07_d03_b04_feedback03	Block_3_senlist.txt	13-Dec-2018_result_f1_3.txt		F2, F4, FC2, FC4, C2, Cz, C1, FC1, FC3, F1, F3, EOGU, EOGD, EOGR, EOGL
		D03	S04	13-Dec-18	Training	p11_v07_d03_b05_training04	Block_4_senlist.txt	-		F2, F4, FC2, FC4, C2, Cz, C1, FC1, FC3, F1, F3, EOGU, EOGD, EOGR, EOGL
		D03	S05	13-Dec-18	Feedback	p11_v07_d03_b06_feedback05	Block_5_senlist.txt	13-Dec-2018_result_f1_5.txt		F2, F4, FC2, FC4, C2, Cz, C1, FC1, FC3, F1, F3, EOGU, EOGD, EOGR, EOGL
		D03	S06	13-Dec-18	*					F2, F4, FC2, FC4, C2, Cz, C1, FC1, FC3, F1, F3, EOGU, EOGD, EOGR, EOGL
		D03	S07	13-Dec-18	*					F2, F4, FC2, FC4, C2, Cz, C1, FC1, FC3, F1, F3, EOGU, EOGD, EOGR, EOGL
		D03	S08	13-Dec-18	*					F2, F4, FC2, FC4, C2, Cz, C1, FC1, FC3, F1, F3, EOGU, EOGD, EOGR, EOGL
		D03	S09	13-Dec-18	Feedback	p11_v07_d03_b11_feedback09	Block_9_senlist.txt	13-Dec-2018_result_f1_9.txt		F2, F4, FC2, FC4, C2, Cz, C1, FC1, FC3, F1, F3, EOGU, EOGD, EOGR, EOGL
	V08	D01	S01	23-Jan-19	Training	p11_v08_d01_b02_Training01	Block_1_senlist.txt	-		F2, F4, FC2, FC4, C4, C2, Cz, C1, FC1, F3, F1, Fz, EOGR, EOGL, EOGUR, EOGDR, EOGUL, EOGDL, EOGDiagR, EOGDiagLU
		D01	S02	23-Jan-19	Training	p11_v08_d01_b03_Training02	Block_2_senlist.txt	-		F2, F4, FC2, FC4, C4, C2, Cz, C1, FC1, F3, F1, Fz, EOGR, EOGL, EOGUR, EOGDR, EOGUL, EOGDL, EOGDiagR, EOGDiagLU
		D01	S03	23-Jan-19	Training	p11_v08_d01_b04_Training03	Block_3_senlist.txt	-		F2, F4, FC2, FC4, C4, C2, Cz, C1, FC1, F3, F1, Fz, EOGR, EOGL, EOGUR, EOGDR, EOGUL, EOGDL, EOGDiagR, EOGDiagLU
		D01	S04	23-Jan-19	Training	p11_v08_d01_b04_Training04	Block_4_senlist.txt	-		F2, F4, FC2, FC4, C4, C2, Cz, C1, FC1, F3, F1, Fz, EOGR, EOGL, EOGUR, EOGDR, EOGUL, EOGDL, EOGDiagR, EOGDiagLU
		D01	S05	23-Jan-19	Feedback	p11_v08_d01_b06_Feedback05	Block_5_senlist.txt	23-Jan-2019_result_f1_5.txt		F2, F4, FC2, FC4, C4, C2, Cz, C1, FC1, F3, F1, Fz, EOGR, EOGL, EOGUR, EOGDR, EOGUL, EOGDL, EOGDiagR, EOGDiagLU
		D01	S06	23-Jan-19	Speller	p11_v08_d01_b07_Speller06	-	23-Jan-2019_result_f1_6.txt	123 trials (w/o trigger 15)	F2, F4, FC2, FC4, C4, C2, Cz, C1, FC1, F3, F1, Fz, EOGR, EOGL, EOGUR, EOGDR, EOGUL, EOGDL, EOGDiagR, EOGDiagLU
		D01	S07	23-Jan-19	Speller	p11_v08_d01_b08_Speller07	-	23-Jan-2019_result_f1_7.txt		F2, F4, FC2, FC4, C4, C2, Cz, C1, FC1, F3, F1, Fz, EOGR, EOGL, EOGUR, EOGDR, EOGUL, EOGDL, EOGDiagR, EOGDiagLU
		D02	S01	24-Jan-19	Training	p11_v08_d02_b02_Training01	Block_1_senlist.txt	-		F2, F4, FC2, FC4, C4, C2, Cz, C1, FC1, F3, F1, Fz, EOGR, EOGL, EOGUR, EOGDR, EOGUL, EOGDL, EOGDiagR, EOGDiagLU
		D02	S02	24-Jan-19	Feedback	p11_v08_d02_b03_Feedback02	Block_2_senlist.txt	24-Jan-2019_result_f1_2.txt		F2, F4, FC2, FC4, C4, C2, Cz, C1, FC1, F3, F1, Fz, EOGR, EOGL, EOGUR, EOGDR, EOGUL, EOGDL, EOGDiagR, EOGDiagLU
		D02	S03	24-Jan-19	Speller	p11_v08_d02_b04_Speller03	-	24-Jan-2019_result_f1_3.txt	33 trials (w/o trigger 15)	F2, F4, FC2, FC4, C4, C2, Cz, C1, FC1, F3, F1, Fz, EOGR, EOGL, EOGUR, EOGDR, EOGUL, EOGDL, EOGDiagR, EOGDiagLU
		D02	S04	24-Jan-19	Speller	p11_v08_d02_b05_Speller04	-	24-Jan-2019_result_f1_4.txt	14 trials (w/o trigger 15)	F2, F4, FC2, FC4, C4, C2, Cz, C1, FC1, F3, F1, Fz, EOGR, EOGL, EOGUR, EOGDR, EOGUL, EOGDL, EOGDiagR, EOGDiagLU
		D02	S05	24-Jan-19	Speller	p11_v08_d02_b06_Speller05	-	24-Jan-2019_result_f1_5.txt	326 trials (w/o trigger 15)	F2, F4, FC2, FC4, C4, C2, Cz, C1, FC1, F3, F1, Fz, EOGR, EOGL, EOGUR, EOGDR, EOGUL, EOGDL, EOGDiagR, EOGDiagLU
		D03	S01	25-Jan-19	Training	p11_v08_d03_b02_training01	Block_1_senlist.txt	-		F2, F4, FC2, FC4, C4, C2, Cz, C1, FC1, F3, F1, Fz, EOGR, EOGL, EOGUR, EOGDR, EOGUL, EOGDL, EOGDiagR, EOGDiagLU
		D03	S02	25-Jan-19	Feedback	p11_v08_d03_b03_feedback02	Block_2_senlist.txt	25-Jan-2019_result_f1_2.txt		F2, F4, FC2, FC4, C4, C2, Cz, C1, FC1, F3, F1, Fz, EOGR, EOGL, EOGUR, EOGDR, EOGUL, EOGDL, EOGDiagR, EOGDiagLU
		D03	S03	25-Jan-19	*					F2, F4, FC2, FC4, C4, C2, Cz, C1, FC1, F3, F1, Fz, EOGR, EOGL, EOGUR, EOGDR, EOGUL, EOGDL, EOGDiagR, EOGDiagLU
		D03	S04	25-Jan-19	*					F2, F4, FC2, FC4, C4, C2, Cz, C1, FC1, F3, F1, Fz, EOGR, EOGL, EOGUR, EOGDR, EOGUL, EOGDL, EOGDiagR, EOGDiagLU
		D03	S05	25-Jan-19	Feedback	p11_v08_d03_b06_feedback05	Block_5_senlist.txt	25-Jan-2019_result_f1_5.txt		F2, F4, FC2, FC4, C4, C2, Cz, C1, FC1, F3, F1, Fz, EOGR, EOGL, EOGUR, EOGDR, EOGUL, EOGDL, EOGDiagR, EOGDiagLU
		D03	S06	25-Jan-19	Feedback	p11_v08_d03_b07_feedback06	Block_6_senlist.txt	25-Jan-2019_result_f1_6.txt	3 Trials, session interrupted	F2, F4, FC2, FC4, C4, C2, Cz, C1, FC1, F3, F1, Fz, EOGR, EOGL, EOGUR, EOGDR, EOGUL, EOGDL, EOGDiagR, EOGDiagLU
		D03	S07	25-Jan-19	Feedback	p11_v08_d03_b08_feedback07	Block_7_senlist.txt	25-Jan-2019_result_f1_7.txt		F2, F4, FC2, FC4, C4, C2, Cz, C1, FC1, F3, F1, Fz, EOGR, EOGL, EOGUR, EOGDR, EOGUL, EOGDL, EOGDiagR, EOGDiagLU
		D03	S08	25-Jan-19	Speller	p11_v08_d03_b09_speller08	-	25-Jan-2019_result_f1_8.txt	26 Trials, intruder Trigger “DCC”	F2, F4, FC2, FC4, C4, C2, Cz, C1, FC1, F3, F1, Fz, EOGR, EOGL, EOGUR, EOGDR, EOGUL, EOGDL, EOGDiagR, EOGDiagLU
		D03	S09	25-Jan-19	Speller	p11_v08_d03_b10_speller09	-	25-Jan-2019_result_f1_9.txt	12 Trials, intruder Trigger “DCC”	F2, F4, FC2, FC4, C4, C2, Cz, C1, FC1, F3, F1, Fz, EOGR, EOGL, EOGUR, EOGDR, EOGUL, EOGDL, EOGDiagR, EOGDiagLU
		D04	S01	26-Jan-19	Training	p11_v08_d04_b02_training	Block_1_senlist.txt	-		F2, F4, FC2, FC4, C4, C2, Cz, C1, FC1, F3, F1, Fz, EOGR, EOGL, EOGUR, EOGDR, EOGUL, EOGDL, EOGDiagR, EOGDiagLU
		D04	S02	26-Jan-19	Feedback	p11_v08_d04_b03_feedback02	Block_2_senlist.txt	26-Jan-2019_result_f1_2.txt		F2, F4, FC2, FC4, C4, C2, Cz, C1, FC1, F3, F1, Fz, EOGR, EOGL, EOGUR, EOGDR, EOGUL, EOGDL, EOGDiagR, EOGDiagLU
		D04	S03	26-Jan-19	Feedback	p11_v08_d04_b04_feedback03	Block_3_senlist.txt	26-Jan-2019_result_f1_3.txt		F2, F4, FC2, FC4, C4, C2, Cz, C1, FC1, F3, F1, Fz, EOGR, EOGL, EOGUR, EOGDR, EOGUL, EOGDL, EOGDiagR, EOGDiagLU
		D04	S04	26-Jan-19	Feedback	p11_v08_d04_b05_feedback04	Block_4_senlist.txt	26-Jan-2019_result_f1_4.txt		F2, F4, FC2, FC4, C4, C2, Cz, C1, FC1, F3, F1, Fz, EOGR, EOGL, EOGUR, EOGDR, EOGUL, EOGDL, EOGDiagR, EOGDiagLU
		D04	S05	26-Jan-19	Feedback	p11_v08_d04_b06_feedback05	Block_5_senlist.txt	26-Jan-2019_result_f1_5.txt		F2, F4, FC2, FC4, C4, C2, Cz, C1, FC1, F3, F1, Fz, EOGR, EOGL, EOGUR, EOGDR, EOGUL, EOGDL, EOGDiagR, EOGDiagLU
	V09	D01	S01	14-Feb-19	Training	p11_v09_d01_b02training01	Block_1_senlist.txt	-		C1, CZ, C2, F4, FC4, F3, FC3, EOGRU, EOGRD, EOGR, EOGL
		D01	S02	14-Feb-19	Training	p11_v09_d01_b03training02	Block_2_senlist.txt	-		C1, CZ, C2, F4, FC4, F3, FC3, EOGRU, EOGRD, EOGR, EOGL
		D01	S03	14-Feb-19	Feedback	p11_v09_d01_b04feedback03	Block_3_senlist.txt	14-Feb-2019_result_f1_3.txt		C1, CZ, C2, F4, FC4, F3, FC3, EOGRU, EOGRD, EOGR, EOGL
		D01	S04	14-Feb-19	Feedback	p11_v09_d01_b05feedback04	Block_4_senlist.txt	14-Feb-2019_result_f1_4.txt		C1, CZ, C2, F4, FC4, F3, FC3, EOGRU, EOGRD, EOGR, EOGL
		D01	S05	14-Feb-19	Feedback	p11_v09_d01_b06feedback06	Block_5_senlist.txt	14-Feb-2019_result_f1_5.txt		C1, CZ, C2, F4, FC4, F3, FC3, EOGRU, EOGRD, EOGR, EOGL
		D01	S06	14-Feb-19	Training	p11_v09_d01_b07training06	Block_6_senlist.txt	-		C1, CZ, C2, F4, FC4, F3, FC3, EOGRU, EOGRD, EOGR, EOGL
		D01	S07	14-Feb-19	Training	p11_v09_d01_b08training07	Block_7_senlist.txt	-		C1, CZ, C2, F4, FC4, F3, FC3, EOGRU, EOGRD, EOGR, EOGL
		D01	S08	14-Feb-19	Feedback	p11_v09_d01_b09feedback08	Block_8_senlist.txt	14-Feb-2019_result_f1_8.txt		C1, CZ, C2, F4, FC4, F3, FC3, EOGRU, EOGRD, EOGR, EOGL
		D01	S09	14-Feb-19	Speller	p11_v09_d01_b10speller09	-	14-Feb-2019_result_f1_9.txt	321 Trials (w/o trigger 15)	C1, CZ, C2, F4, FC4, F3, FC3, EOGRU, EOGRD, EOGR, EOGL
		D02	S01	15-Feb-19	Training	p11_v09_d02_b02training01	Block_1_senlist.txt	-		C1, CZ, C2, F4, FC4, F3, FC3, EOGRU, EOGRD, EOGR, EOGL
		D02	S02	15-Feb-19	Feedback	p11_v09_d02_b03feedback02	Block_2_senlist.txt	15-Feb-2019_result_f1_2.txt		C1, CZ, C2, F4, FC4, F3, FC3, EOGRU, EOGRD, EOGR, EOGL
		D02	S03	15-Feb-19	Feedback	p11_v09_d02_b03feedback03	Block_3_senlist.txt	15-Feb-2019_result_f1_3.txt		C1, CZ, C2, F4, FC4, F3, FC3, EOGRU, EOGRD, EOGR, EOGL
		D02	S04	15-Feb-19	Speller	p11_v09_d02_b05speller04	-	15-Feb-2019_result_f1_4.txt		C1, CZ, C2, F4, FC4, F3, FC3, EOGRU, EOGRD, EOGR, EOGL
		D02	S05	15-Feb-19	Speller	p11_v09_d02_b06speller05	-	15-Feb-2019_result_f1_5.txt	321 Trials (w/o trigger 15)	C1, CZ, C2, F4, FC4, F3, FC3, EOGRU, EOGRD, EOGR, EOGL
	V10	D01	S01	16-Mar-19	Training	p11_v10_d01_b02_training01	Block_1_senlist.txt	-		F4, FC2, FC4, C2, Cz, C1, FC1, FC3, F3, EOGU, EOGD, EOGR, EOGL, EOGDUR, EOGDDL
		D01	S02	16-Mar-19	Training	p11_v10_d01_b03_training02	Block_2_senlist.txt	-		F4, FC2, FC4, C2, Cz, C1, FC1, FC3, F3, EOGU, EOGD, EOGR, EOGL, EOGDUR, EOGDDL
		D01	S03	16-Mar-19	Training	p11_v10_d01_b04_training03	Block_3_senlist.txt	-		F4, FC2, FC4, C2, Cz, C1, FC1, FC3, F3, EOGU, EOGD, EOGR, EOGL, EOGDUR, EOGDDL
		D01	S04	16-Mar-19	Training	p11_v10_d01_b05_training04	Block_4_senlist.txt	-		F4, FC2, FC4, C2, Cz, C1, FC1, FC3, F3, EOGU, EOGD, EOGR, EOGL, EOGDUR, EOGDDL
		D01	S05	16-Mar-19	Training	p11_v10_d01_b06_training05	Block_5_senlist.txt	-		F4, FC2, FC4, C2, Cz, C1, FC1, FC3, F3, EOGU, EOGD, EOGR, EOGL, EOGDUR, EOGDDL
		D01	S06	16-Mar-19	Training	p11_v10_d01_b07_training06	Block_6_senlist.txt	-		F4, FC2, FC4, C2, Cz, C1, FC1, FC3, F3, EOGU, EOGD, EOGR, EOGL, EOGDUR, EOGDDL
		D01	S07	16-Mar-19	Feedback	p11_v10_d01_b08_feedback07	Block_7_senlist.txt	16-Mar-2019_result_f1_7.txt		F4, FC2, FC4, C2, Cz, C1, FC1, FC3, F3, EOGU, EOGD, EOGR, EOGL, EOGDUR, EOGDDL
		D01	S08	16-Mar-19	Feedback	p11_v10_d01_b09_feedback08	Block_8_senlist.txt	16-Mar-2019_result_f1_8.txt		F4, FC2, FC4, C2, Cz, C1, FC1, FC3, F3, EOGU, EOGD, EOGR, EOGL, EOGDUR, EOGDDL
		D01	S09	16-Mar-19	Speller	p11_v10_d01_b10_speller09	-	16-Mar-2019_result_f1_9.txt		F4, FC2, FC4, C2, Cz, C1, FC1, FC3, F3, EOGU, EOGD, EOGR, EOGL, EOGDUR, EOGDDL
		D01	S10	16-Mar-19	Speller	p11_v10_d01_b11_speller10	-	16-Mar-2019_result_f1_10.txt	5 Trials, intruder Triggers after the end of session	F4, FC2, FC4, C2, Cz, C1, FC1, FC3, F3, EOGU, EOGD, EOGR, EOGL, EOGDUR, EOGDDL
		D01	S11	16-Mar-19	Training	p11_v10_d01_b12_training11	Block_11_senlist.txt	-		F4, FC2, FC4, C2, Cz, C1, FC1, FC3, F3, EOGU, EOGD, EOGR, EOGL, EOGDUR, EOGDDL
		D01	S12	16-Mar-19	Feedback	p11_v10_d01_b13_feedback12	Block_12_senlist.txt	16-Mar-2019_result_f1_12.txt		F4, FC2, FC4, C2, Cz, C1, FC1, FC3, F3, EOGU, EOGD, EOGR, EOGL, EOGDUR, EOGDDL

A detailed list of the sequence of visits (V), days (D), sessions (S) and the dates in which each session of the study was recorded, and their precise correspondence with the given raw file, and other files associated with the particular sessions.

The sixth column from left to right shows the type of session, namely, Training, Feedback or Speller session. Sessions indicated with (*) were recorded for a study outside the scope of this research. In sessions indicated with (Motor Attempt), the experimenters asked the patient to perform Motor Attempt task, instead of eye movement, to answer to the questions. The columns seven, eight and nine show the precise names of the raw, sentence list and result list files, respectively, corresponding to the particular session. Column ten indicates some special remarks to consider in the files. Column eleven indicates the labels of the EOG and EEG electrodes (10-20 system) used in the day of recording. Raw files of the speller sessions which were interrupted by the experimenter to take care of the needs of the patient misses the end trigger, i.e., the trigger number 15, as written in the Remark column. During V08 and V10, because of diminishing oculomotor activity, we increased the number of electrodes around eyes to increase the sensitivity of our system, because of which, we placed four extra electrodes namely EOGUL, EOGUD, EOGDiagR and EOGDiagUL.

**Online-only Table 2:** Session to Raw File-Sentence List-Result List Correspondence for P13.

Patient	Visit	Day	Session	Date	Type of Session	Raw File Name (*.eeg)	Sentence List Name (*.txt)	Result List Name (*.txt)	Remark	Electrodes
P13	V01	D01	S01	20-Jun-18	Training	d1_0001	Block_1_senlist.txt	—		EOGU, EOGD, EOGR, EOGL, AF4, F4, AF3, F3, Cz, C1, C2
		D01	S02	20-Jun-18	Training	d1_0002	Block_2_senlist.txt	-		EOGU, EOGD, EOGR, EOGL, AF4, F4, AF3, F3, Cz, C1, C2
		D01	S03	20-Jun-18	Training	d1_0003	Block_3_senlist.txt	-		EOGU, EOGD, EOGR, EOGL, AF4, F4, AF3, F3, Cz, C1, C2
		D02	S01	21-Jun-18	Training	d2_0001	(Lost-overwritten) Block_1_senlist.txt		It was overwriten by a restarting of the system	EOGU, EOGD, EOGR, EOGL, AF4, F4, AF3, F3, Cz, C1, C2
		D02	S02	21-Jun-18	Training	d2_0002	Block_2_senlist.txt			EOGU, EOGD, EOGR, EOGL, AF4, F4, AF3, F3, Cz, C1, C2
		D02	S03	21-Jun-18	Feedback	d2_0003_fb1	Block_3_senlist.txt	(Corrupted) 21-Jun-2018_result_f1_3.txt	Too many predictions in the Result List	EOGU, EOGD, EOGR, EOGL, AF4, F4, AF3, F3, Cz, C1, C2
		D02	S04	21-Jun-18	Feedback	d2_0004_fb2	Block_1_senlist.txt	21-Jun-2018_result_f1_1.txt		EOGU, EOGD, EOGR, EOGL, AF4, F4, AF3, F3, Cz, C1, C2
		D03	S01	22-Jun-18	Training	d3_0001	Block_1_senlist.txt	-		EOGU, EOGD, EOGR, EOGL, AF4, F4, AF3, F3, Cz, C1, C2
		D03	S02	22-Jun-18	Feedback	d3_0002_fb1	Block_2_senlist.txt	22-Jun-2018_result_f1_2.txt		EOGU, EOGD, EOGR, EOGL, AF4, F4, AF3, F3, Cz, C1, C2
		D03	S02	22-Jun-18	*					EOGU, EOGD, EOGR, EOGL, AF4, F4, AF3, F3, Cz, C1, C2
		D03	S03	22-Jun-18	*					EOGU, EOGD, EOGR, EOGL, AF4, F4, AF3, F3, Cz, C1, C2
	V02	D01	S01	15-Oct-18	Training	p13_v02_d01_b01_training01	Block_1_senlist.txt	-		EOGU, EOGD, EOGR, EOGL, AF4, F4, AF3, F3, Cz, C1, C2, Fz, C4, EMG1, EMG2
		D01	S02	15-Oct-18	Training	p13_v02_d01_b02_training02	Block_2_senlist.txt	-		EOGU, EOGD, EOGR, EOGL, AF4, F4, AF3, F3, Cz, C1, C2, Fz, C4, EMG1, EMG2
		D01	S03	15-Oct-18	Training	p13_v02_d01_b03_training03	Block_3_senlist.txt	-		EOGU, EOGD, EOGR, EOGL, AF4, F4, AF3, F3, Cz, C1, C2, Fz, C4, EMG1, EMG2
		D01	S04	15-Oct-18	Feedback	p13_v02_d01_b04_feedback01	Block_4_senlist.txt	15-Oct-2018_result_f1_4.txt		EOGU, EOGD, EOGR, EOGL, AF4, F4, AF3, F3, Cz, C1, C2, Fz, C4, EMG1, EMG2
		D01	S05	15-Oct-18	Feedback	p13_v02_d01_b05_feedback02	Block_5_senlist.txt	15-Oct-2018_result_f1_5.txt		EOGU, EOGD, EOGR, EOGL, AF4, F4, AF3, F3, Cz, C1, C2, Fz, C4, EMG1, EMG2
		D01	S06	15-Oct-18	*	p13_v02_d01_b06_open01	Block_6_senlist.txt	15-Oct-2018_result_f1_6.txt		EOGU, EOGD, EOGR, EOGL, AF4, F4, AF3, F3, Cz, C1, C2, Fz, C4, EMG1, EMG2
		D02	S01	16-Oct-18	Training	p13_v02_d02_b2_training1	Block_1_senlist.txt	-		EOGU, EOGD, EOGR, EOGL, AF4, F4, AF3, F3, Cz, C1, C2, Fz, C4, EMG1, EMG2
		D02	S02	16-Oct-18	Feedback	p13_v02_d02_b3_feedback1	Block_2_senlist.txt	16-Oct-2018_result_f1_2.txt		EOGU, EOGD, EOGR, EOGL, AF4, F4, AF3, F3, Cz, C1, C2, Fz, C4, EMG1, EMG2
		D02	S03	16-Oct-18	Feedback	p13_v02_d02_b4_feedback2	Block_3_senlist.txt	16-Oct-2018_result_f1_3.txt		EOGU, EOGD, EOGR, EOGL, AF4, F4, AF3, F3, Cz, C1, C2, Fz, C4, EMG1, EMG2
		D02	S04	16-Oct-18	*					EOGU, EOGD, EOGR, EOGL, AF4, F4, AF3, F3, Cz, C1, C2, Fz, C4, EMG1, EMG2
		D02	S05	16-Oct-18	*					EOGU, EOGD, EOGR, EOGL, AF4, F4, AF3, F3, Cz, C1, C2, Fz, C4, EMG1, EMG2
		D02	S06	16-Oct-18	*					EOGU, EOGD, EOGR, EOGL, AF4, F4, AF3, F3, Cz, C1, C2, Fz, C4, EMG1, EMG2
		D03	S01	17-Oct-18	Training	p13_v02_d03_b02_training01	Block_1_senlist.txt	-		EOGU, EOGD, EOGR, EOGL, AF4, F4, AF3, F3, Cz, C1, C2, Fz, C4, EMG1, EMG2
		D03	S02	17-Oct-18	Feedback	p13_v02_d03_b03_feedback01	Block_2_senlist.txt	17-Oct-2018_result_f1_2.txt		EOGU, EOGD, EOGR, EOGL, AF4, F4, AF3, F3, Cz, C1, C2, Fz, C4, EMG1, EMG2
		D03	S03	17-Oct-18	Speller	p13_v02_d03_b04_speller01	-	17-Oct-2018_result_f1_3.txt	20 complete trials (w/o trigger 15) (Bug in code)	EOGU, EOGD, EOGR, EOGL, AF4, F4, AF3, F3, Cz, C1, C2, Fz, C4, EMG1, EMG2
		D03	S04	17-Oct-18	Speller	p13_v02_d03_b05_speller02	-	17-Oct-2018_result_f1_4.txt	(Bug in code)	EOGU, EOGD, EOGR, EOGL, AF4, F4, AF3, F3, Cz, C1, C2, Fz, C4, EMG1, EMG2
		D03	S05	17-Oct-18	Speller	p13_v02_d03_b06_speller03	-	17-Oct-2018_result_f1_5.txt		EOGU, EOGD, EOGR, EOGL, AF4, F4, AF3, F3, Cz, C1, C2, Fz, C4, EMG1, EMG2
		D03	S06	17-Oct-18	Speller	p13_v02_d03_b07_speller04	-	17-Oct-2018_result_f1_6.txt		EOGU, EOGD, EOGR, EOGL, AF4, F4, AF3, F3, Cz, C1, C2, Fz, C4, EMG1, EMG2
		D03	S07	17-Oct-18	Speller	p13_v02_d03_b08_speller05	17-Oct-2018_result_f1_7.txt	68 complete trials (w/o trigger 15)		EOGU, EOGD, EOGR, EOGL, AF4, F4, AF3, F3, Cz, C1, C2, Fz, C4, EMG1, EMG2
	V03	D01	-	19-Feb-19	*		-	-		EOGRU, EOGRD, EOGRH, EOGLH, C1, CZ, C2, AF3, F3, AF4, F4, EMG1, EMG2
		D01	S01	19-Feb-19	Training	p13_v03_d01_b01_tr01	Block_1_senlist.txt	-		EOGRU, EOGRD, EOGRH, EOGLH, C1, CZ, C2, AF3, F3, AF4, F4, EMG1, EMG2
		D01	S02	19-Feb-19	Training	p13_v03_d01_b02_fb01	Block_2_senlist.txt	-		EOGRU, EOGRD, EOGRH, EOGLH, C1, CZ, C2, AF3, F3, AF4, F4, EMG1, EMG2
		D01	S03	19-Feb-19	Training	p13_v03_d01_b02_fb03	Block_3_senlist.txt	-		EOGRU, EOGRD, EOGRH, EOGLH, C1, CZ, C2, AF3, F3, AF4, F4, EMG1, EMG5
		D01	S04	19-Feb-19	Feedback	p13_v03_d01_b02_fb04	Block_4_senlist.txt	19-Feb-2019_result_f1_4.txt		EOGRU, EOGRD, EOGRH, EOGLH, C1, CZ, C2, AF3, F3, AF4, F4, EMG1, EMG2
		D01	S05	19-Feb-19	Feedback	Contained in the V03D01S04	Block_5_senlist.txt	19-Feb-2019_result_f1_5.txt	Only 10 trials	EOGRU, EOGRD, EOGRH, EOGLH, C1, CZ, C2, AF3, F3, AF4, F4, EMG1, EMG2
		D02	S01	20-Feb-19	Training	p13_v03_d02_b01_tr01	Block_1_senlist.txt	-		EOGRU, EOGRD, EOGRH, EOGLH, C1, CZ, C2, AF3, F3, AF4, F4, EMG1, EMG2
		D02	S02	20-Feb-19	Training	p13_v03_d02_b02_tr02	Block_2_senlist.txt	-		EOGRU, EOGRD, EOGRH, EOGLH, C1, CZ, C2, AF3, F3, AF4, F4, EMG1, EMG2
		D02	S03	20-Feb-19	Feedback	p13_v03_d01_fb01	Block_3_senlist.txt	20-Feb-2019_result_f1_3.txt		EOGRU, EOGRD, EOGRH, EOGLH, C1, CZ, C2, AF3, F3, AF4, F4, EMG1, EMG2
		D02	S04	20-Feb-19	Feedback	p13_v03_d02_b04_fb02	Block_4_senlist.txt	20-Feb-2019_result_f1_4.txt		EOGRU, EOGRD, EOGRH, EOGLH, C1, CZ, C2, AF3, F3, AF4, F4, EMG1, EMG2
		D02	S05	20-Feb-19	Speller	p13_v03_d02_b05_sp01	-	20-Feb-2019_result_f1_5.txt	88 complete trials (w/o trigger 15)	EOGRU, EOGRD, EOGRH, EOGLH, C1, CZ, C2, AF3, F3, AF4, F4, EMG1, EMG2
		D02	S06	20-Feb-19	Speller	p13_v03_d02_b06_sp02	-	20-Feb-2019_result_f1_6.txt		EOGRU, EOGRD, EOGRH, EOGLH, C1, CZ, C2, AF3, F3, AF4, F4, EMG1, EMG2
		D02	S07	20-Feb-19	Speller	p13_v03_d02_b07_sp03	-	20-Feb-2019_result_f1_7.txt		EOGRU, EOGRD, EOGRH, EOGLH, C1, CZ, C2, AF3, F3, AF4, F4, EMG1, EMG2
		D02	S08	20-Feb-19	Speller	p13_v03_d02_b08_sp04	-	20-Feb-2019_result_f1_8.txt		EOGRU, EOGRD, EOGRH, EOGLH, C1, CZ, C2, AF3, F3, AF4, F4, EMG1, EMG2
		D03	S01	21-Feb-19	Feedback	p13_v03_d03_b01_fb01	Block_1_senlist.txt	(Lost) 21-Feb-2019_result_f1_1.txt	Lost Result File	EOGRU, EOGRD, EOGRH, EOGLH, C1, CZ, C2, AF3, F3, AF4, F4, EMG1, EMG2
		D03	S02	21-Feb-19	Speller	p13_v03_d03_b02_sp01	-	21-Feb-2019_result_f1_2.txt	298 complete trials (w/o trigger 15)	EOGRU, EOGRD, EOGRH, EOGLH, C1, CZ, C2, AF3, F3, AF4, F4, EMG1, EMG2
		D04	S01	22-Feb-19	Feedback	p13_v03_d04_b01_fb01	Block_1_senlist.txt	(Lost) 22-Feb-2019_result_f1_1.txt	Lost Result File	EOGRU, EOGRD, EOGRH, EOGLH, C1, CZ, C2, AF3, F3, AF4, F4, EMG1, EMG2
		D04	S02	22-Feb-19	Speller	p13_v03_d04_b02_sp01	-	22-Feb-2019_result_f1_2.txt	78 complete trials (w/o trigger 15)	EOGRU, EOGRD, EOGRH, EOGLH, C1, CZ, C2, AF3, F3, AF4, F4, EMG1, EMG2
		D04	S03	22-Feb-19	Speller	p13_v03_d04_b03_sp02	-	22-Feb-2019_result_f1_3.txt		EOGRU, EOGRD, EOGRH, EOGLH, C1, CZ, C2, AF3, F3, AF4, F4, EMG1, EMG2
	V04	D01	S01	28-May-19	Training	p13_v04_d01_b0001	Block_1_senlist			AF3, F3, C1, Cz, C2, AF4, F4, EOGRU, EOGRD, EOGRH, EOGLH
		D01	S02	28-May-19	Training	p13_v04_d01_b0003	Block_2_senlist			AF3, F3, C1, Cz, C2, AF4, F4, EOGRU, EOGRD, EOGRH, EOGLH
		D01	S03	28-May-19	Feedback	p13_v04_d01_bf0003	Block_3_senlist	28-May-2019_result_f1_3**	7 initial triggers from a false start	AF3, F3, C1, Cz, C2, AF4, F4, EOGRU, EOGRD, EOGRH, EOGLH
		D01	S04	28-May-19	Feedback	p13_v04_d01_bf0004	Block_4_senlist	28-May-2019_result_f1_4**		AF3, F3, C1, Cz, C2, AF4, F4, EOGRU, EOGRD, EOGRH, EOGLH
		D01	S05	28-May-19	Feedback	p13_v04_d01_bf005	Block_5_senlist	28-May-2019_result_f1_5**		AF3, F3, C1, Cz, C2, AF4, F4, EOGRU, EOGRD, EOGRH, EOGLH
		D02	S01	29-May-19	Training	p13_v04_d02_b0001	Block_1_senlist.txt			AF3, F3, C1, Cz, C2, AF4, F4, EOGRU, EOGRD, EOGRH, EOGLH
		D02	S02	29-May-19	Training	p13_v04_d02_b0002	Block_2_senlist.txt			AF3, F3, C1, Cz, C2, AF4, F4, EOGRU, EOGRD, EOGRH, EOGLH
		D02	S03	29-May-19	Training	p13_v04_d02_btf0003	Block_3_senlist.txt			AF3, F3, C1, Cz, C2, AF4, F4, EOGRU, EOGRD, EOGRH, EOGLH
		D02	S04	29-May-19	Training	p13_v04_d02_btf0004	Block_4_senlist.txt			AF3, F3, C1, Cz, C2, AF4, F4, EOGRU, EOGRD, EOGRH, EOGLH
		D02	S05	29-May-19	Feedback	p13_v04_d02_bAF0005	Block_5_senlist.txt	29-May-2019_result_f1_5.txt		AF3, F3, C1, Cz, C2, AF4, F4, EOGRU, EOGRD, EOGRH, EOGLH
		D02	S06	29-May-19	Feedback	p13_v04_d02_baf0010	Block_6_senlist.txt	29-May-2019_result_f1_6.txt		AF3, F3, C1, Cz, C2, AF4, F4, EOGRU, EOGRD, EOGRH, EOGLH
		D02	S07	29-May-19	Feedback	p13_v04_d02_baf0011	Block_7_senlist.txt	29-May-2019_result_f1_7.txt		AF3, F3, C1, Cz, C2, AF4, F4, EOGRU, EOGRD, EOGRH, EOGLH
		D02	S08	29-May-19	Speller	p13_v04_d02_bSP0012	29-May-2019_result_f1_8.txt			AF3, F3, C1, Cz, C2, AF4, F4, EOGRU, EOGRD, EOGRH, EOGLH
		D02	S09	29-May-19	Speller	p13_v04_d02_bsp0013	29-May-2019_result_f1_9.txt			AF3, F3, C1, Cz, C2, AF4, F4, EOGRU, EOGRD, EOGRH, EOGLH
		D03	S01	30-May-19	Training	p13_v04_d03_b0001	Block_1_senlist.txt			AF3, F3, C1, Cz, C2, AF4, F4, EOGRU, EOGRD, EOGRH, EOGLH
		D03	S02	30-May-19	Training	p13_v04_d03_b0002	Block_2_senlist.txt			AF3, F3, C1, Cz, C2, AF4, F4, EOGRU, EOGRD, EOGRH, EOGLH
		D03	S03	30-May-19	Training	p13_v04_d03_btf0003	Block_3_senlist.txt			AF3, F3, C1, Cz, C2, AF4, F4, EOGRU, EOGRD, EOGRH, EOGLH
		D03	S04	30-May-19	Feedback	p13_v04_d03_baf0004	Block_4_senlist.txt	30-May-2019_result_f1_4.txt		AF3, F3, C1, Cz, C2, AF4, F4, EOGRU, EOGRD, EOGRH, EOGLH
		D03	S05	30-May-19	Speller	p13_v04_d03_bSP0005	30-May-2019_result_f1_5.txt			AF3, F3, C1, Cz, C2, AF4, F4, EOGRU, EOGRD, EOGRH, EOGLH
		D03	S06	30-May-19	Speller	p13_v04_d03_bSp0006	30-May-2019_result_f1_6.txt			AF3, F3, C1, Cz, C2, AF4, F4, EOGRU, EOGRD, EOGRH, EOGLH
		D03	S07	30-May-19	Speller	p13_v04_d03_bSP0007	30-May-2019_result_f1_7.txt			AF3, F3, C1, Cz, C2, AF4, F4, EOGRU, EOGRD, EOGRH, EOGLH
		D03	S08	30-May-19	Speller	p13_v04_d03_bsp0008	30-May-2019_result_f1_8.txt			AF3, F3, C1, Cz, C2, AF4, F4, EOGRU, EOGRD, EOGRH, EOGLH
		D04	S01	31-May-19	Training	p13_v04_d04_b0001	Block_1_senlist.txt			AF3, F3, C1, Cz, C2, AF4, F4, EOGRU, EOGRD, EOGRH, EOGLH
		D04	S02	31-May-19	Training	p13_v04_d04_b0002	Block_2_senlist.txt			AF3, F3, C1, Cz, C2, AF4, F4, EOGRU, EOGRD, EOGRH, EOGLH
		D04	S03	31-May-19	Training	p13_v04_d04_btf0003	Block_3_senlist.txt			AF3, F3, C1, Cz, C2, AF4, F4, EOGRU, EOGRD, EOGRH, EOGLH
		D04	S04	31-May-19	Speller	p13_v04_d04_bsp0004	31-May-2019_result_f1_4.txt			AF3, F3, C1, Cz, C2, AF4, F4, EOGRU, EOGRD, EOGRH, EOGLH
		D04	S05	31-May-19	Speller	p13_v04_d04_bsp0005	31-May-2019_result_f1_5.txt			AF3, F3, C1, Cz, C2, AF4, F4, EOGRU, EOGRD, EOGRH, EOGLH
		D04	S06	31-May-19	Speller	p13_v04_d04_bsp0006	31-May-2019_result_f1_6.txt	179 complete trials (w/o trigger 15), extra triggers at the end		AF3, F3, C1, Cz, C2, AF4, F4, EOGRU, EOGRD, EOGRH, EOGLH

A detailed list of the sequence of visits (V), days (D), sessions (S) and the dates in which each session of the study was recorded, and their precise correspondence with the given raw file, and other files associated with the particular sessions.

The sixth column from left to right shows the type of session, namely, Training, Feedback or Speller session. Sessions indicated with (*) were recorded for a study outside the scope of this research. The columns seven, eight and nine show the precise names of the raw, sentence list and result list files, respectively, corresponding to the particular session. The elements in column nine indicated with (**), are coded oppositely as they are indicated in the Methods section of the paper, i.e., listing the predicted results as “0” if the answer was recognized as “no”, “1” if the answer was recognized as “yes. Column ten indicates some special remarks to consider in the files. Column eleven indicates the labels of the EOG and EEG electrodes (10-20 system) used in the day of recording. Raw files of the speller sessions which were interrupted by the experimenter to take care of the needs of the patient misses the end trigger, i.e., the trigger number 15, as written in the Remark column.

**Online-only Table 3:** Session to Raw File-Sentence List-Result List Correspondence for P15.

Patient	Visit	Day	Session	Date	Type of Session	Raw File Name (*.eeg)	Sentence List Name (*.txt)	Result List Name (*.txt)	Remark	Electrodes
P15	V01	D01	S01	25-Feb-19	Training	p15_v01_d01_b01_training01	Block_1_senlist.txt	-		AFF2, FFC2, C1, Cz, C2, AFF1, FCC1, EOGRU, EOGRD, EOGRH, EOGLH
		D01	S02	25-Feb-19	Training	p15_v01_d01_b02_feedback01	Block_2_senlist.txt	25-Feb-2019_result_f1_2.txt		AFF2, FFC2, C1, Cz, C2, AFF1, FCC1, EOGRU, EOGRD, EOGRH, EOGLH
		D01	S03	25-Feb-19	Training	p15_v01_d01_b03_feedback02	Block_3_senlist.txt	25-Feb-2019_result_f1_3.txt		AFF2, FFC2, C1, Cz, C2, AFF1, FCC1, EOGRU, EOGRD, EOGRH, EOGLH
		D01	S04	25-Feb-19	Feedback	p15_v01_d01_b04_feedback03	Block_4_senlist.txt	25-Feb-2019_result_f1_4.txt		AFF2, FFC2, C1, Cz, C2, AFF1, FCC1, EOGRU, EOGRD, EOGRH, EOGLH
		D02	S01	26-Feb-19	Feedback	(Lost) p15_v01_d02_b01_feedback	Block_1_senlist.txt	26-Feb-2019_result_f1_1.txt	Lost raw file	AF3, F3, C1, Cz, C2, AF4, F4, EOGU, EOGD, EOGR, EOGL
		D02	S02	26-Feb-19	Feedback	p15_v01_d02_b02_feedback	Block_2_senlist.txt	26-Feb-2019_result_f1_2.txt	Feature modified w/baseline correction	AF3, F3, C1, Cz, C2, AF4, F4, EOGU, EOGD, EOGR, EOGL
		D03	S01	27-Feb-19	Training	p15_v01_d03_b0001_training01	Block_1_senlist.txt	-		AF3, F3, C1, Cz, C2, AF4, F4, EOGU, EOGD, EOGR, EOGL
		D03	S02	27-Feb-19	Feedback	p15_v01_d03_b02_feedback01	Block_2_senlist.txt	27-Feb-2019_result_f1_2.txt		AF3, F3, C1, Cz, C2, AF4, F4, EOGU, EOGD, EOGR, EOGL
		D03	S03	27-Feb-19	Speller	p15_v01_d03_b03_speller01	-	27-Feb-2019_result_f1_3.txt	7 trials (w/o trigger 15) (only “yes”)	AF3, F3, C1, Cz, C2, AF4, F4, EOGU, EOGD, EOGR, EOGL
		D03	S04	27-Feb-19	Speller	p15_v01_d03_b04_speller02_ok	-	27-Feb-2019_result_f1_4.txt	16 trials (w/o trigger 15), 5 trials (w/o trigger 15)	AF3, F3, C1, Cz, C2, AF4, F4, EOGU, EOGD, EOGR, EOGL
		D03	S05	27-Feb-19	Feedback	p15_v01_d03_b05_feedback02	Block_5_senlist.txt	27-Feb-2019_result_f1_5.txt	Only ten questions	AF3, F3, C1, Cz, C2, AF4, F4, EOGU, EOGD, EOGR, EOGL
		D03	S06	27-Feb-19	Speller	p15_v01_d03_b06_speller03	-	27-Feb-2019_result_f1_6.txt	5 trials (w/o trigger 15)	AF3, F3, C1, Cz, C2, AF4, F4, EOGU, EOGD, EOGR, EOGL
		D03	S07	27-Feb-19	Speller	p15_v01_d03_b07_spelller04	-	27-Feb-2019_result_f1_7.txt	16 trials (w/o trigger 15 (only “yes”)	AF3, F3, C1, Cz, C2, AF4, F4, EOGU, EOGD, EOGR, EOGL
		D04	S01	28-Feb-19	Training	p15_v01_d04_b01_training	Block_1_senlist.txt	-		AF3, F3, C1, Cz, C2, AF4, F4, EOGU, EOGD, EOGR, EOGL
		D04	S02	28-Feb-19	Feedback	p15_v01_d04_b02_feedback	Block_2_senlist.txt	28-Feb-2019_result_f1_2.txt		AF3, F3, C1, Cz, C2, AF4, F4, EOGU, EOGD, EOGR, EOGL
		D04	S03	28-Feb-19	*					AF3, F3, C1, Cz, C2, AF4, F4, EOGU, EOGD, EOGR, EOGL
		D04	S04	28-Feb-19	*					AF3, F3, C1, Cz, C2, AF4, F4, EOGU, EOGD, EOGR, EOGL
		D04	S05	28-Feb-19	Speller	p15_v01_d04_b05_speller01	-	28-Feb-2019_result_f1_5.txt		AF3, F3, C1, Cz, C2, AF4, F4, EOGU, EOGD, EOGR, EOGL
		D04	S06	28-Feb-19	Speller	p15_v01_d04_b06_speller02	-	28-Feb-2019_result_f1_6.txt	112 trials (w/o trigger 15)	AF3, F3, C1, Cz, C2, AF4, F4, EOGU, EOGD, EOGR, EOGL
		D04	S07	28-Feb-19	Speller	p15_v01_d04_b07_speller03	-	28-Feb-2019_result_f1_7.txt	9 trials (w/o trigger 15) (only “yes”)	AF3, F3, C1, Cz, C2, AF4, F4, EOGU, EOGD, EOGR, EOGL
		D04	S08	28-Feb-19	Speller	p15_v01_d04_b08_speller04	-	28-Feb-2019_result_f1_8.txt	4 trials (w/o trigger 15) (only “yes”)	AF3, F3, C1, Cz, C2, AF4, F4, EOGU, EOGD, EOGR, EOGL
		D04	S09	28-Feb-19	Speller	p15_v01_d04_b09_speller05	-	28-Feb-2019_result_f1_9.txt	8 trials (w/o trigger 15) (only “yes”)	AF3, F3, C1, Cz, C2, AF4, F4, EOGU, EOGD, EOGR, EOGL
	V02	D01	S01	25-Jun-19	Training	p15_v02_d01_b01_training	Block_1_senlist.txt	-		F3, AF3, F4, AF4, C1, Cz, C2, EOGU, EOGD, EOGR, EOGL
		D01	S02	25-Jun-19	Training	p15_v02_d01_b02_training02	Block_2_senlist.txt	-		F3, AF3, F4, AF4, C1, Cz, C2, EOGU, EOGD, EOGR, EOGL
		D01	S03	25-Jun-19	Feedback	p15_v02_d01_b03_feedback	Block_3_senlist.txt	25-Jun-2019_result_f1_3.txt		F3, AF3, F4, AF4, C1, Cz, C2, EOGU, EOGD, EOGR, EOGL
		D01	S04	25-Jun-19	Feedback	p15_v02_d01_b04_feedback	Block_4_senlist.txt	25-Jun-2019_result_f1_4.txt		F3, AF3, F4, AF4, C1, Cz, C2, EOGU, EOGD, EOGR, EOGL
		D01	S05	25-Jun-19	Feedback	p15_v02_d01_b05_feedback	Block_5_senlist.txt	25-Jun-2019_result_f1_5.txt		F3, AF3, F4, AF4, C1, Cz, C2, EOGU, EOGD, EOGR, EOGL
		D01	S06	25-Jun-19	Speller	p15_v02_d01_b06_speller	-	25-Jun-2019_result_f1_6.txt	28 trials (w/o trigger 15) (No selection made)	F3, AF3, F4, AF4, C1, Cz, C2, EOGU, EOGD, EOGR, EOGL
		D01	S07	25-Jun-19	Speller	p15_v02_d01_b07_speller	-	25-Jun-2019_result_f1_7.txt	67 trials (w/o trigger 15)	F3, AF3, F4, AF4, C1, Cz, C2, EOGU, EOGD, EOGR, EOGL
		D02	S01	26-Jun-19	Feedback	p15_v02_d02_b02_training	Block_1_senlist.txt	26-Jun-2019_result_f1_1.txt		AF3, F3, AF4, F4, C1, Cz, C2, EOGU, EOGD, EOGR, EOGL
		D02	S02	26-Jun-19	Speller	p15_v02_d02_b03_speller	-	26-Jun-2019_result_f1_2.txt	282 trials (w/o trigger 15)	AF3, F3, AF4, F4, C1, Cz, C2, EOGU, EOGD, EOGR, EOGL
		D02	S03	26-Jun-19	Speller	p15_v02_d02_b04_speller	-	26-Jun-2019_result_f1_3.txt		AF3, F3, AF4, F4, C1, Cz, C2, EOGU, EOGD, EOGR, EOGL
		D02	S04	26-Jun-19	Speller	p15_v02_d02_b05_speller	-	26-Jun-2019_result_f1_4.txt	476 trials (w/o trigger 15)	AF3, F3, AF4, F4, C1, Cz, C2, EOGU, EOGD, EOGR, EOGL
		D03	S01	27-Jun-19	Feedback	p15_v02_d03_b02_feedback	Block_1_senlist.txt	27-Jun-2019_result_f1_1.txt	Session stopped	AF3, F3, AF4, F4, C1, Cz, C2, EOGU, EOGD, EOGR, EOGL
		D03	S02	27-Jun-19	Feedback	p15_v02_d03_b03_feedback	Block_2_senlist.txt	27-Jun-2019_result_f1_2.txt		AF3, F3, AF4, F4, C1, Cz, C2, EOGU, EOGD, EOGR, EOGL
		D03	S03	27-Jun-19	Feedback	p15_v02_d03_b04_feedback	Block_3_senlist.txt	27-Jun-2019_result_f1_3.txt		AF3, F3, AF4, F4, C1, Cz, C2, EOGU, EOGD, EOGR, EOGL
		D03	S04	27-Jun-19	Feedback	p15_v02_d03_b05_feedback	Block_4_senlist.txt	27-Jun-2019_result_f1_4.txt		AF3, F3, AF4, F4, C1, Cz, C2, EOGU, EOGD, EOGR, EOGL
		D03	S05	27-Jun-19	Feedback	p15_v02_d03_b06_feedback	Block_5_senlist.txt	27-Jun-2019_result_f1_5.txt		AF3, F3, AF4, F4, C1, Cz, C2, EOGU, EOGD, EOGR, EOGL
		D03	S06	27-Jun-19	Feedback	p15_v02_d03_b07_feedback	Block_6_senlist.txt	27-Jun-2019_result_f1_6.txt		AF3, F3, AF4, F4, C1, Cz, C2, EOGU, EOGD, EOGR, EOGL
		D03	S07	27-Jun-19	Speller	p15_v02_d03_b08_speller	-	27-Jun-2019_result_f1_7.txt		AF3, F3, AF4, F4, C1, Cz, C2, EOGU, EOGD, EOGR, EOGL
		D03	S08	27-Jun-19	Speller	p15_v02_d03_b09_speller	-	27-Jun-2019_result_f1_8.txt	2 trials (w/o trigger 15) (No selection made)	AF3, F3, AF4, F4, C1, Cz, C2, EOGU, EOGD, EOGR, EOGL
		D03	S09	28-Jun-19	Speller	p15_v02_d03_b10_speller	-	27-Jun-2019_result_f1_9.txt	2 trials (w/o trigger 15) (No selection made)	AF3, F3, AF4, F4, C1, Cz, C2, EOGU, EOGD, EOGR, EOGL
		D03	S10	27-Jun-19	*		-			AF3, F3, AF4, F4, C1, Cz, C2, EOGU, EOGD, EOGR, EOGL
		D03	S11	27-Jun-19	*		-			AF3, F3, AF4, F4, C1, Cz, C2, EOGU, EOGD, EOGR, EOGL
		D03	S12	27-Jun-19	*		-			AF3, F3, AF4, F4, C1, Cz, C2, EOGU, EOGD, EOGR, EOGL
		D03	S13	27-Jun-19	*		-			AF3, F3, AF4, F4, C1, Cz, C2, EOGU, EOGD, EOGR, EOGL
		D03	S14	27-Jun-19	Speller	p15_v02_d03_b15_speller	-	27-Jun-2019_result_f1_14.txt	48 trials (w/o trigger 15) (only “no”)	AF3, F3, AF4, F4, C1, Cz, C2, EOGU, EOGD, EOGR, EOGL

A detailed list of the sequence of visits (V), days (D), sessions (S) and the dates in which each session of the study was recorded, and their precise correspondence with the given raw file, and other files associated with the particular sessions.

The sixth column from left to right shows the type of session, namely, Training, Feedback or Speller session. Sessions indicated with (*) were recorded for a study outside the scope of this research. The columns seven, eight and nine show the precise names of the raw, sentence list and result list files, respectively, corresponding to the particular session. Column ten indicates some special remarks to consider in the files. Column eleven indicates the labels of the EOG and EEG electrodes (10-20 system) used in the day of recording. Raw files of the speller sessions which were interrupted by the experimenter to take care of the needs of the patient misses the end trigger, i.e., the trigger number 15, as written in the Remark column.

**Online-only Table 4:** Session to Raw File-Sentence List-Result List Correspondence for P16.

Patient	Visit	Day	Session	Date	Type of Session	Raw File Name (*.eeg)	Sentence List Name (*.txt)	Result List Name (*.txt)	Remark	Electrodes
P16	V01	D01	S01	4-Mar-19	Training	p16_v01_d01_b0001_tr01	Block_1_senlist.txt	-		AF3, F3, C1, Cz, C2, AF4, F4, EOGU, EOGD, EOGR, EOGL
		D01	S02	4-Mar-19	Training	p16_v01_d01_b0002_fb01	Block_2_senlist.txt	-		AF3, F3, C1, Cz, C2, AF4, F4, EOGU, EOGD, EOGR, EOGL
		D01	S03	4-Mar-19	Training	p16_v01_d01_b0003_fb02	Block_3_senlist.txt	-		AF3, F3, C1, Cz, C2, AF4, F4, EOGU, EOGD, EOGR, EOGL
		D01	S04	4-Mar-19	Training	p16_v01_d01_b0004_fb03	Block_4_senlist.txt	-		AF3, F3, C1, Cz, C2, AF4, F4, EOGU, EOGD, EOGR, EOGL
		D01	S05	4-Mar-19	Feedback	p16_v01_d01_b0005_fb04	Block_5_senlist.txt	04-Mar-2019_result_f1_5.txt		AF3, F3, C1, Cz, C2, AF4, F4, EOGU, EOGD, EOGR, EOGL
		D02	S01	5-Mar-19	*			-	False start, it was deleted	AF3, F3, C1, Cz, C2, AF4, F4, EOGU, EOGD, EOGR, EOGL
		D02	S02	5-Mar-19	Feedback	p16_v01_d01_b0002_fb02	Block_2_senlist.txt	05-Mar-2019_result_f1_2.txt		AF3, F3, C1, Cz, C2, AF4, F4, EOGU, EOGD, EOGR, EOGL
		D02	S03	5-Mar-19	Feedback	p16_v01_d01_b0003_fb03	Block_3_senlist.txt	05-Mar-2019_result_f1_3.txt		AF3, F3, C1, Cz, C2, AF4, F4, EOGU, EOGD, EOGR, EOGL
		D02	S04	5-Mar-19	Feedback	p16_v01_d01_b0004_fb04	Block_4_senlist.txt	05-Mar-2019_result_f1_4.txt		AF3, F3, C1, Cz, C2, AF4, F4, EOGU, EOGD, EOGR, EOGL
		D02	S05	5-Mar-19	*	p16_v01_d01_b0005_sp01				AF3, F3, C1, Cz, C2, AF4, F4, EOGU, EOGD, EOGR, EOGL
		D02	S06	5-Mar-19	*	p16_v01_d01_b0006_sp02				AF3, F3, C1, Cz, C2, AF4, F4, EOGU, EOGD, EOGR, EOGL
		D02	S07	5-Mar-19	Speller	p16_v01_d01_b0007_sp03	-	05-Mar-2019_result_f1_7.txt	34 trials (trigger 15 and triggers of interrupted trial)	AF3, F3, C1, Cz, C2, AF4, F4, EOGU, EOGD, EOGR, EOGL
		D02	S08	5-Mar-19	Speller	p16_v01_d01_b0008_sp04	-	05-Mar-2019_result_f1_8.txt	62 trials (w/o trigger 15)	AF3, F3, C1, Cz, C2, AF4, F4, EOGU, EOGD, EOGR, EOGL
		D03	S01	6-Mar-19	Training	p16_v01_d03_b0001_fb01	Block_1_senlist.txt	-		AF3, F3, C1, Cz, C2, AF4, F4, EOGU, EOGD, EOGR, EOGL
		D03	S02	6-Mar-19	Feedback	p16_v01_d03_b0002_fb02	Block_2_senlist.txt	06-Mar-2019_result_f1_2.txt		AF3, F3, C1, Cz, C2, AF4, F4, EOGU, EOGD, EOGR, EOGL
		D03	S03	6-Mar-19	Feedback	p16_v01_d03_b0003_fb03	Block_3_senlist.txt	06-Mar-2019_result_f1_3.txt		AF3, F3, C1, Cz, C2, AF4, F4, EOGU, EOGD, EOGR, EOGL
		D03	S04	6-Mar-19	Feedback	p16_v01_d03_b0004_fb04	Block_4_senlist.txt	06-Mar-2019_result_f1_4.txt		AF3, F3, C1, Cz, C2, AF4, F4, EOGU, EOGD, EOGR, EOGL
		D03	S05	6-Mar-19	*					AF3, F3, C1, Cz, C2, AF4, F4, EOGU, EOGD, EOGR, EOGL
		D03	S06	6-Mar-19	*					AF3, F3, C1, Cz, C2, AF4, F4, EOGU, EOGD, EOGR, EOGL
		D03	S07	6-Mar-19	Training	p16_v01_d03_b0007_tr01	Block_7_senlist.txt	-		AF3, F3, C1, Cz, C2, AF4, F4, EOGU, EOGD, EOGR, EOGL
		D03	S08	6-Mar-19	Feedback	p16_v01_d03_b0008_fb05	Block_8_senlist.txt	06-Mar-2019_result_f1_8.txt	Only ten questions	AF3, F3, C1, Cz, C2, AF4, F4, EOGU, EOGD, EOGR, EOGL
		D03	S09	6-Mar-19	Training	p16_v01_d03_b0009_fb06	Block_9_senlist.txt	-		AF3, F3, C1, Cz, C2, AF4, F4, EOGU, EOGD, EOGR, EOGL
		D03	S10	6-Mar-19	Training	p16_v01_d03_b0010_fb07	Block_10_senlist.txt	-		AF3, F3, C1, Cz, C2, AF4, F4, EOGU, EOGD, EOGR, EOGL
		D04	S01	7-Mar-19	Training	p16_v01_d04_b0001_tr01	Block_1_senlist.txt	-		AF3, F3, C1, Cz, C2, AF4, F4, EOGU, EOGD, EOGR, EOGL
		D04	S02	7-Mar-19	Training	p16_v01_d04_b0002_tr02	Block_2_senlist.txt	-		AF3, F3, C1, Cz, C2, AF4, F4, EOGU, EOGD, EOGR, EOGL
		D04	S03	7-Mar-19	Training	p16_v01_d04_b0003_tr03	Block_3_senlist.txt	-		AF3, F3, C1, Cz, C2, AF4, F4, EOGU, EOGD, EOGR, EOGL
		D04	S04	7-Mar-19	Training	p16_v01_d04_b0004_tr04	Block_4_senlist.txt	-		AF3, F3, C1, Cz, C2, AF4, F4, EOGU, EOGD, EOGR, EOGL
		D04	S05	7-Mar-19	Feedback	p16_v01_d04_b0005_fb03	Block_5_senlist.txt	07-Mar-2019_result_f1_5.txt		AF3, F3, C1, Cz, C2, AF4, F4, EOGU, EOGD, EOGR, EOGL
		D04	S06	7-Mar-19	Feedback	p16_v01_d04_b0006_fb04	Block_6_senlist.txt	07-Mar-2019_result_f1_6.txt		AF3, F3, C1, Cz, C2, AF4, F4, EOGU, EOGD, EOGR, EOGL
		D04	S07	7-Mar-19	Speller	p16_v01_d04_b0006_sp01	-	07-Mar-2019_result_f1_7.txt		AF3, F3, C1, Cz, C2, AF4, F4, EOGU, EOGD, EOGR, EOGL
		D04	S08	7-Mar-19	*		-		Start of less than 1 sec. Useless and deleted	AF3, F3, C1, Cz, C2, AF4, F4, EOGU, EOGD, EOGR, EOGL
		D05	S01	7-Mar-19	Training	p16_v01_d05_b0001_tr01	Block_1_senlist.txt	-		AF3, F3, C1, Cz, C2, AF4, F4, EOGU, EOGD, EOGR, EOGL
		D05	S02	7-Mar-19	Training	p16_v01_d05_b0002_tr02	Block_2_senlist.txt	-		AF3, F3, C1, Cz, C2, AF4, F4, EOGU, EOGD, EOGR, EOGL
		D05	S03	7-Mar-19	Training	p16_v01_d05_b0003_tr03	Block_3_senlist.txt	-		AF3, F3, C1, Cz, C2, AF4, F4, EOGU, EOGD, EOGR, EOGL
		D05	S04	7-Mar-19	Feedback	p16_v01_d05_b0003_fb02	Block_4_senlist.txt	08-Mar-2019_result_f1_4.txt		AF3, F3, C1, Cz, C2, AF4, F4, EOGU, EOGD, EOGR, EOGL
		D05	S05	7-Mar-19	Speller	p16_v01_d05_b0004_sp01	-	08-Mar-2019_result_f1_5.txt		AF3, F3, C1, Cz, C2, AF4, F4, EOGU, EOGD, EOGR, EOGL
		D05	S06	7-Mar-19	Speller	p16_v01_d05_b0005_sp02	-	08-Mar-2019_result_f1_6.txt		AF3, F3, C1, Cz, C2, AF4, F4, EOGU, EOGD, EOGR, EOGL
	V02	D01	S01	20-May-19	Training	p06_v02_d0001_b01_tr01	Block_1_senlist.txt	-		AF3, F3, C1, Cz, C2, AF4, F4, EOGU, EOGD, EOGR, EOGL
		D01	S02	20-May-19	Training	p06_v02_d0001_b02_tr02	Block_2_senlist.txt	-		AF3, F3, C1, Cz, C2, AF4, F4, EOGU, EOGD, EOGR, EOGL
		D01	S03	20-May-19	Training	p06_v02_d0001_b03_tr03	Block_3_senlist.txt	-		AF3, F3, C1, Cz, C2, AF4, F4, EOGU, EOGD, EOGR, EOGL
		D01	S04	20-May-19	Training	p06_v02_d0001_b04_tr04	Block_4_senlist.txt	-		AF3, F3, C1, Cz, C2, AF4, F4, EOGU, EOGD, EOGR, EOGL
		D01	S05	20-May-19	Training	p06_v02_d0001_b05_tr05	Block_5_senlist.txt	-		AF3, F3, C1, Cz, C2, AF4, F4, EOGU, EOGD, EOGR, EOGL
		D01	S06	20-May-19	Training	p06_v02_d0001_b06_tr06	Block_6_senlist.txt	-		AF3, F3, C1, Cz, C2, AF4, F4, EOGU, EOGD, EOGR, EOGL
		D01	S07	20-May-19	Training	p06_v02_d0001_b07_tr07	Block_7_senlist.txt	-		AF3, F3, C1, Cz, C2, AF4, F4, EOGU, EOGD, EOGR, EOGL
		D02	S01	21-May-19	Training	p06_v02_d02_b00001_tr01	Block_1_senlist.txt	-		AF3, F3, C1, Cz, C2, AF4, F4, EOGU, EOGD, EOGR, EOGL
		D02	S02	21-May-19	Training	p06_v02_d02_b00002_tr02	Block_2_senlist.txt	-		AF3, F3, C1, Cz, C2, AF4, F4, EOGU, EOGD, EOGR, EOGL
		D02	S03	21-May-19	Feedback	p06_v02_d02_b00003_fb01	Block_3_senlist.txt	(Corrupted) 21-May-2019_result_f1_3.txt	Response list was corrupted. Online PA impossible to verify.	AF3, F3, C1, Cz, C2, AF4, F4, EOGU, EOGD, EOGR, EOGL
		D02	S04	21-May-18	Feedback	p06_v02_d02_b00004_fb02	Block_4_senlist.txt	21-May-2019_result_f1_4.txt		AF3, F3, C1, Cz, C2, AF4, F4, EOGU, EOGD, EOGR, EOGL
		D02	S05	21-May-19	Feedback	p06_v02_d02_b00005_fb03	Block_5_senlist.txt	21-May-2019_result_f1_5.txt		AF3, F3, C1, Cz, C2, AF4, F4, EOGU, EOGD, EOGR, EOGL
		D02	S06	21-May-19	Speller	p06_v02_d02_b00005_sp01	-	21-May-2019_result_f1_6.txt		AF3, F3, C1, Cz, C2, AF4, F4, EOGU, EOGD, EOGR, EOGL
		D03	S01	22-May-19	*		Block_1_senlist.txt	-	False start. Useless and deleted	AF3, F3, C1, Cz, C2, AF4, F4, EOGU, EOGD, EOGR, EOGL
		D03	S02	22-May-19	Training	p06_v02_d03_b00002_tr02	Block_2_senlist.txt	-		AF3, F3, C1, Cz, C2, AF4, F4, EOGU, EOGD, EOGR, EOGL
		D03	S03	22-May-19	Feedback	p06_v02_d03_b00003_fb01	Block_3_senlist.txt	22-May-2019_result_f1_3.txt		AF3, F3, C1, Cz, C2, AF4, F4, EOGU, EOGD, EOGR, EOGL
		D03	S04	22-May-19	Feedback	p06_v02_d03_b00004_fb02	Block_4_senlist.txt	22-May-2019_result_f1_4.txt		AF3, F3, C1, Cz, C2, AF4, F4, EOGU, EOGD, EOGR, EOGL
		D03	S05	22-May-19	Speller	p06_v02_d03_b00005_sp01	-	22-May-2019_result_f1_5.txt		AF3, F3, C1, Cz, C2, AF4, F4, EOGU, EOGD, EOGR, EOGL
		D04	S01	23-May-19	Training	p06_v02_d04_b00001_tr01	Block_1_senlist.txt	-		AF3, F3, C1, Cz, C2, AF4, F4, EOGU, EOGD, EOGR, EOGL
		D04	S02	23-May-19	Training	p06_v02_d04_b00002_tr02	Block_2_senlist.txt	-		AF3, F3, C1, Cz, C2, AF4, F4, EOGU, EOGD, EOGR, EOGL
		D04	S03	23-May-19	Feedback	p06_v02_d04_b00003_fb01	Block_3_senlist.txt	23-May-2019_result_f1_3.txt		AF3, F3, C1, Cz, C2, AF4, F4, EOGU, EOGD, EOGR, EOGL
		D04	S04	23-May-19	Feedback	p06_v02_d04_b00004_fb02	Block_4_senlist.txt	23-May-2019_result_f1_4.txt		AF3, F3, C1, Cz, C2, AF4, F4, EOGU, EOGD, EOGR, EOGL
		D04	S05	23-May-19	Feedback	p06_v02_d04_b00005_fb03	Block_5_senlist.txt	23-May-2019_result_f1_5.txt		AF3, F3, C1, Cz, C2, AF4, F4, EOGU, EOGD, EOGR, EOGL
		D04	S06	23-May-19	Feedback	p06_v02_d04_b00006_fb04	Block_6_senlist.txt	23-May-2019_result_f1_6.txt		AF3, F3, C1, Cz, C2, AF4, F4, EOGU, EOGD, EOGR, EOGL
		D04	S07	23-May-19	Feedback	p06_v02_d04_b00007_fb05	Block_7_senlist.txt	23-May-2019_result_f1_7.txt		AF3, F3, C1, Cz, C2, AF4, F4, EOGU, EOGD, EOGR, EOGL
		D04	S08	23-May-19	Feedback	p06_v02_d04_b00008_fb06	Block_8_senlist.txt	23-May-2019_result_f1_8.txt		AF3, F3, C1, Cz, C2, AF4, F4, EOGU, EOGD, EOGR, EOGL
		D04	S09	23-May-19	Speller	p06_v02_d04_b00009_fb07	-	23-May-2019_result_f1_9.txt		AF3, F3, C1, Cz, C2, AF4, F4, EOGU, EOGD, EOGR, EOGL

A detailed list of the sequence of visits (V), days (D), sessions (S) and the dates in which each session of the study was recorded, and their precise correspondence with the given raw file, and other files associated with the particular sessions.

The sixth column from left to right shows the type of session, namely, Training, Feedback or Speller session. Sessions indicated with (*) were recorded for a study outside the scope of this research. The columns seven, eight and nine show the precise names of the raw, sentence list and result list files, respectively, corresponding to the particular session. Column ten indicates some special remarks to consider in the files. Column eleven indicates the labels of the EOG and EEG electrodes (10-20 system) used in the day of recording. Raw files of the speller sessions which were interrupted by the experimenter to take care of the needs of the patient misses the end trigger, i.e., the trigger number 15, as written in the Remark column.
