# Supplementary material for: A dataset of EEG and EOG from an auditory EOG-based communication system for patients in locked-in state
Source: Sci Data. 2021 Jan 11;8:8. doi: 10.1038/s41597-020-00789-4 (PMC7801642; doi:10.1038/s41597-020-00789-4)
Supplement: Supplementary file 1 — Supplementary material [file 41597_2020_789_MOESM1_ESM.docx]

**Data Use Agreement**

**Institute of Medical Psychology and Behavioral Neurobiology, University of Tübingen, Germany**

1. Statement of the Agreement

This “Data Use Agreement” (Agreement) is made as of the last dated signature below by, and between Dr. Ujwal Chaudhary as the owner and “Custodian of the Data” (Custodian), and the “Recipient of the Data” (Recipient). The purpose of this Agreement is 1) to satisfy certain obligations of the Recipient, to ensure the integrity and confidentiality of the “Patients and their relatives or caretakers” (Patients) from whom the data has been acquired with proper consent, and 2) to guarantee ethical and good use of the Data itself and any “Personal Information” from the Patients.

Any Data and Personal Information has been collected with permission of the Patients and their relatives or caretakers, as established in a proper signed Consent Form, kept by the Custodian. As a consequence, the Custodian is the keeper of the Data and Personal Information and contact of the Patients.

1. Origin of the Data

The Data has been previously used in the publications:

1. "A dataset of EEG and EOG from an auditory EOG-based communication system for patients in locked-In state" by Andres Jaramillo-Gonzalez, Shize Wu, Alessandro Tonin, Aygul Rana, Majid Khalili-Ardali, Niels Birbaumer, and Ujwal Chaudhary, to be published in the journal *Scientific Data*, and
2. “Auditory Electrooculogram-based Communication System for ALS Patients in Transition from Locked-in to Complete Locked-in State” by Alessandro Tonin, Andres Jaramillo-Gonzalez, Aygul Rana, Majid Khalili-Ardali, Niels Birbaumer, and Ujwal Chaudhary, published in the journal *Scientific Reports*, **10,**8452 (2020). <https://doi.org/10.1038/s41598-020-65333-1>

Nevertheless, Data was used and shared without revealing or using Personal Information in them. Consequently, the Data itself can be defined as a “Limited Data Set”, i.e., Data that excludes the following direct identifiers of the Patient: 1. Names; 2. Postal address information, other than town or city, state, and zip code; 3. Telephone numbers or Fax numbers; 4. Electronic mail addresses; 5. Social security numbers; 6. Medical record numbers; 7. Health plan beneficiary numbers; 8. Account numbers; 9. Certificate/license numbers; 10. Vehicle identifiers and serial numbers, including license plate numbers; 11. Device identifiers and serial numbers; 12. Web Universal Resource Locators (URLs); 13. Internet Protocol (IP) address numbers; 14. Biometric identifiers, including finger and voice prints; and 15. Full face photographic images and any comparable images.

Nevertheless, as explained in the aforementioned publications, the Data is linked to “Digital Audio Files” (Audios) that reveal Personal Information from the Patients and their relatives or caretakers. Considering that, for research purposes, Audios can be useful to validate, extend, or derivate the scientific clinical research already performed on the Data, the Custodian can consider sharing these Audios with a Recipient, by complying with the requested in this Agreement.

1. Conditions of the Agreement

WHEREAS, the Custodian has the approval to share the Audios with the Recipient, and

WHEREAS, Recipient agrees to receive the Audio Files on the following terms and conditions, and WHEREAS, in consideration of the foregoing and other good and valuable consideration, the receipt and sufficiency of which are hereby acknowledged, Custodian and the Recipient agree that:

- The Recipient is permitted to use the Audios in conjunction with the Limited Data Set, or any Personal Information, only for scientific, academic, educational, and non-commercial research. No further use is permitted hereunder and commercial use is strictly prohibited.
- The Recipient will properly acknowledge and reference the Custodian and his scientific research Group as the owner and Custodian of the Limited Data Set, the Audios, and Personal Information, and any scientific protocol depending on exposing the Patient’s Personal information must be discussed with the Custodian, to preserve the Patient’s right of privacy.
- The Recipient recognizes the Custodian as the primary contact with the Patient, and the Recipient recognizes the contribution of the Patients and/or Caretakers in sharing Data and Personal Information, and consequently...
- any request of Personal Information beyond the mere Audios must be first exposed to and discussed with the Custodian, to conjunctly evaluate the need and pertinence of exposing Personal Information, only with the purposes mentioned above.
- The Recipient recognizes and respects the right of privacy of the Patient and relatives and caretakers, and consequently will avoid to trace back their identities from the Audios information, or to perform any unnecessary contact with them if Personal Information is shared.
- The Recipient will not disclose or share the Audios or any Personal Information given by the Custodian, and mandatorily, any intention in sharing the Audios or personal Information with third parties must be discussed with the Custodian and if necessary, with the Patient and/or relatives and caretakers.
- In receiving the Audios or Personal Information, the Recipient will turn into a custodian of the information and the right of privacy of the Patient and his/her relatives and caretakers.
- In the case of being authorized in sharing the Audios or Personal Information, the Recipient will turn into a Custodian of the data, committed to this same Data Agreement.

Dr. Ujwal Chaudhary and the authorized representatives of the Recipient party have signed this Agreement as set forth below.

**Custody of the Data/project leader:**

| Dr. Ujwal Chaudhary | Date: |
| --- | --- |
| Institutional description: | Signature: |
| Contact: |  |

**Recipient of the Data:**

| Name: | Date: |
| --- | --- |
| Title: | Signature: |
| Adscription: | Institutional stamp: |
| Address: |  |
| Personal contact: |  |
